# Supplementary material for: Evaluating the impact of maternal health care policy on stillbirth and perinatal mortality in Ghana; a mixed method approach using two rounds of Ghana demographic and health survey data sets and qualitative design technique
Source: PLoS One. 2022 Sep 29;17(9):e0274573. doi: 10.1371/journal.pone.0274573 (PMC9521900; doi:10.1371/journal.pone.0274573)
Supplement: S1 File — (DOCX) [file pone.0274573.s001.docx]

UNIVERSITY OF GHANA

**School of Public Health**

DATE: ___/___/___

**IN-DEPTH INTERVIEW GUIDE**

**Target participants:**

- Doctors
- Midwives

**Introduction:**

My name is __________________________I’m a PhD student of the University of Ghana School of Public Health (UG-SPH). Thanks, a great deal for agreeing to talk to me for about 1hour. As I explained, your identity in this study will be not be disclosed. However, to enable me keep to your comments and use it for data transcription, I will like to record our interaction. I’m particular interested in your experience as far as the free policy is concern.

**Open-ended questions:**

***NB:*** *Follow ups on each question will be allowed, where necessary for clarity purposes and certainty.*

1. How long have you been working in this facility and how is your work related to pregnant women?

….............................................................................................................................................................................................................................................................................................

1. You may have heard there is free access to health care for expectant mothers. How do you understand the free maternal health care that is being provided for pregnant women?

….............................................................................................................................................................................................................................................................................................

1. What financial/material commitments are there for pregnant women who come for antenatal care or facility delivery in this hospital/health centre/clinic?

……………………………………………………………………………………………………………………………………………………………………………………………

1. What are the challenges that confront you in relations to the services that you provide to pregnant women in this hospital/health centre/clinic?

……………………………………………………………………………………………………………………………………………………………………………………………

1. In your experience in this facility, how would you describe pregnant women and mother’s situation with respect to access to services and stillbirth?

……………………………………………………………………………………………………………………………………………………………………………………………

1. In what way does the FMHCP improve or hurt stillbirth?

………………………………………………………………………………………………………………………………………………………………………………………………

1. What suggestion do you have to improve the care of pregnant women in your facility?

………………………………………………………………………………………………………………………………………………………………………………………………

1. Does the policy interfere/hurt your professional practice and how if so?

……………………………………………………………………………………………………………………………………………………………………………………………

1. Thank you for agreeing to talk to me. The responses you gave will help me report findings that will contribute to knowledge as well support to quality maternal and child health care.

DATE: ___/___/___

**Participants:**

- Pregnant women

**Introduction**

There is no right or wrong answer, therefore, everyone is encouraged to participate. Since everyone’s opinion matters in this discussion, I encourage you to speak up your mind whether you agree with the one sitting next to you or not.

**Ice breaker**

1. **Amina’s experience in Labour- (Fiction)**

Amina was rushed into the labour unit of her hometown hospital. Her mother in-law who accompanied her was told to go and buy soap and other things that Amina will require for safe delivery. When she returned, the midwives again told her that Amina will also need some drugs to manage her pain. Thus, she spent the rest of the money on her to buy the drugs and thankfully, Amina gave birth to a bouncy baby girl. There was no difficulty in the delivery of the baby so the doctor discharged Amina the following day and told her that, she needed to pay sanitation fee of Gh20.00 to the hospital cashier before she can go home. Her mother in-law, confused, managed to explain that she had spent all the money on her and only had lorry fare for them to go home.

*Probes*

- 1. Have you experienced anything similar to what Amina’s experienced?
  2. Can you share your experience with us especially about the staff and your pregnancy?
  3. Were you told to pay money or buy other items in this hospital or any other hospital while you were pregnant and accessing care?
  4. What items were you told to buy and how much did you pay for it?

**Closing**

Thank you once again for agreeing to be part of this study.

**Thank you.**

UNIVERSITY OF GHANA

**School of Public Health**

KII Guide

**Target:**  Directors (Expert Informant)

**Introduction**

Thank you for agreeing to talk to me on my study. Our discussion is to gather your expert view on the ‘free’ maternal health care policy implementation and perinatal deaths in the region. As captured on the participant’s information sheet, responses elicited from you are for academic purposes only. This interview is expected to end within **1 hour.**

**The questions guiding our interaction are as follows:**

1. This region has a very good record for Antenatal care and skilled delivery utilization. What would you say has been the reason?

………………………………………………………………………………………………………

1. Mothers sometimes pay for antenatal care at the lab particularly, while accessing antenatal care and also pay for/or buy sanitary items at labour wards.
   1. Has this come to your attention in this facility?....................................................................
   2. How do you think this phenomenon might contribute to affect care and stillbirth/or early neonatal deaths in the region?................................................................................................
2. The issue of drug shortages has often been mentioned in other jurisdiction of care vis a vis the NHIS. What is the situation here and how has this affected your work? ………………………………………………………………………………………………………
3. Despite the ‘free’ policy, there are concerns that mothers still have challenges accessing care when pregnant. What is your observation, working as director for GHS?
4. What is the stillbirth/perinatal mortality situation in this region?
5. Do you think the mothers cooperate with your care demands and how does this affect your outcomes?
6. What is your comments in terms of midwifery practice and the management of stillbirth?.............................................................................................................................................
7. What will be your recommendation, going forward to better the ‘free’ policy and maternal health care and perinatal mortality outcomes in general?

**NB:** The results of this study may be shared with relevant authorities to inform decision making aimed at improving perinatal health care outcomes in Ghana.

**Thank you.**
