# Supplementary material for: Evaluating the impact of maternal health care policy on stillbirth and perinatal mortality in Ghana; a mixed method approach using two rounds of Ghana demographic and health survey data sets and qualitative design technique
Source: PLoS One. 2022 Sep 29;17(9):e0274573. doi: 10.1371/journal.pone.0274573 (PMC9521900; doi:10.1371/journal.pone.0274573)
Supplement: Attachment — Submitted filename: PONE-D-21-35524_reviewer.pdf [file pone.0274573.s003.pdf]

PLOS ONE

Evaluating the impact of maternal health care policy on stillbirth and perinatal mortality in Ghana: a mix method approach using two rounds of Ghana demographic and health survey data sets and qualitative design technique

--Manuscript Draft--

**Manuscript Number:**	PONE-D-21-35524
**Article Type:**	Research Article
**Full Title:**	Evaluating the impact of maternal health care policy on stillbirth and perinatal mortality in Ghana: a mix method approach using two rounds of Ghana demographic and health survey data sets and qualitative design technique
**Short Title:**	Evaluating the impact of maternal health care policy on stillbirth and perinatal mortality in Ghana
**Corresponding Author:**	John Azaare, Mphil<br>University for Development Studies<br>Tamale, GHANA
**Keywords:**	Impact; stillbirth; perinatal mortality; maternal health care; contextual factors.
**Abstract:**	Abstract<br><br>Background: Stillbirth and perinatal mortality indices appear to be declining at a slow pace in Ghana despite the 'free' maternal health care policy intervention since 2008, hence, raising concerns as to whether Ghana is on course to achieving the World Health Organization target of 12 per 1000 live births by the year 2030.<br><br>Purpose: In this study, we compared stillbirth and perinatal mortality outcomes between 2008 and 2014 using Ghana demographic and health survey data set since the 'free' policy inception. We further explored any contextual factors underpinning the policy operation to find explanation to the current situation relative to stillbirth and perinatal mortality.<br><br>Methods: The study adopted a mix method approach, first using two rounds of Ghana Demographic and Health Survey data sets, 2008 and 2014 as baseline and end line respectively. We constructed outcome variables of stillbirth and perinatal mortality from the under 5 mortality variables of two rounds of GDHS (n=487), and then estimated the overall rates and proportion of stillbirth and perinatal mortality for the two rounds. We then analysed for association using Poisson and multiple logistics regressions and also with negative binomial regression models to test for sensitivity, while adjusting for; maternal age, parity, area of residence, educational status, marital status, wealth index, and region as independent covariates, statistically significance, p< 0.05. We also conducted in-depth interviews for service providers and held focus group discussions with pregnant women to examine any contextual factors to draw conclusions and lessons for policy makers.<br><br>Results: The results show that stillbirth rate increased in 2014 by 2 per 1000 live births, while perinatal mortality rate declined within the same period by 4 per 1000 live births, compared 2008. However, both stillbirth and perinatal mortality increased in proportion between 2008 and 2014 by 14.4% and 10.8%, respectively. The analysis of association found that pregnant women were 1.64 times more likely to record stillbirth; aOR: 1.64; 95% [CI: 1.02, 2.65] and 2.04 times more likely to record perinatal mortality; aOR: 2.04; 95% [CI: 1.28, 3.25] among the 'free' maternal health care policy group, and these were statistically significant, p< 0.041; p< 0.003, respectively.<br><br>The qualitative study found that, routine medicines such as folic acid and multivitamine were in intermittently in short supply forcing private purchase by pregnant women to augment their routine requirement. Also, the study found that pregnant women in labour took local concoction as oxytocin ostensibly to fast track the labour process and inadvertently causing complications of uterine rapture increasing the likelihood of stillbirths.

Type text here

Powered by Editorial Manager® and ProduXion Manager® from Aries Systems Corporation

	Conclusion: Even though perinatal mortality rate decline overall between 2008 and 2014, the proportion of stillbirth and perinatal death is still on the rise, thus, the 'free' policy intervention has yet to translate to the desired decline in stillbirths since its inception in 2008. Shortage of medicine commodities, inadequate monitoring of labour process coupled with pregnant women intake of traditional herbs are probably some explanations to the current and perhaps influencing stillbirth outcomes.
Order of Authors:	John Azaare, Mphil
	Patricia Akweongo, PhD
	Genevieve Cecelia Aryeetey, PhD
	Duah Dwomoh
Additional Information:	
Question	Response
**Financial Disclosure**<br><br>Enter a financial disclosure statement that describes the sources of funding for the work included in this submission. Review the submission guidelines for detailed requirements. View published research articles from *PLOS ONE* for specific examples.<br><br>This statement is required for submission and **will appear in the published article** if the submission is accepted. Please make sure it is accurate.<br><br>**Unfunded studies**<br>Enter: *The author(s) received no specific funding for this work.*<br><br>**Funded studies**<br>Enter a statement with the following details:<br>• Initials of the authors who received each award<br>• Grant numbers awarded to each author<br>• The full name of each funder<br>• URL of each funder website<br>• Did the sponsors or funders play any role in the study design, data collection and analysis, decision to publish, or preparation of the manuscript?<br>• **NO** - Include this sentence at the end of your statement: *The funders had no role in study design, data collection and analysis, decision to publish, or preparation of the manuscript.*<br>• **YES** - Specify the role(s) played.<br><br>* typeset	The authors received no specific funding for this work.
**Competing Interests**	The authors have declared that no competing interest exist.

Use the instructions below to enter a competing interest statement for this submission. On behalf of all authors, disclose any competing interests that could be perceived to bias this work—acknowledging all financial support and any other relevant financial or non-financial competing interests.<br><br>This statement is required for submission and **will appear in the published article** if the submission is accepted. Please make sure it is accurate and that any funding sources listed in your Funding Information later in the submission form are also declared in your Financial Disclosure statement.<br><br>View published research articles from *PLOS ONE* for specific examples.<br><br>**NO authors have competing interests**<br><br>Enter: *The authors have declared that no competing interests exist.*<br><br>**Authors with competing interests**<br><br>Enter competing interest details beginning with this statement:<br><br>*I have read the journal's policy and the authors of this manuscript have the following competing interests: [insert competing interests here]*<br><br>* typeset	
**Ethics Statement**<br><br>Enter an ethics statement for this submission. This statement is required if the study involved:<br><br>• Human participants<br>• Human specimens or tissue<br>• Vertebrate animals or cephalopods<br>• Vertebrate embryos or tissues	This study received ethical approval from the Ghana Health Service Ethics Review Committee with no. GHS-ERC 002/04/19. Also, entrée to health facilities were formally granting in writing. All study participants consented in writing, to be part of the qualitative interviews and focus group discussion.

• Field research<br><br>Write "N/A" if the submission does not require an ethics statement.<br><br>General guidance is provided below. Consult the submission guidelines for detailed instructions. **Make sure that all information entered here is included in the Methods section of the manuscript.**	

Powered by Editorial Manager® and ProduXion Manager® from Aries Systems Corporation

**Format for specific study types**<br><br>**Human Subject Research (involving human participants and/or tissue)**<br>• Give the name of the institutional review board or ethics committee that approved the study<br>• Include the approval number and/or a statement indicating approval of this research<br>• Indicate the form of consent obtained (written/oral) or the reason that consent was not obtained (e.g. the data were analyzed anonymously)<br><br>**Animal Research (involving vertebrate animals, embryos or tissues)**<br>• Provide the name of the Institutional Animal Care and Use Committee (IACUC) or other relevant ethics board that reviewed the study protocol, and indicate whether they approved this research or granted a formal waiver of ethical approval<br>• Include an approval number if one was obtained<br>• If the study involved *non-human primates*, add *additional details* about animal welfare and steps taken to ameliorate suffering<br>• If anesthesia, euthanasia, or any kind of animal sacrifice is part of the study, include briefly which substances and/or methods were applied<br><br>**Field Research**<br><br>Include the following details if this study involves the collection of plant, animal, or other materials from a natural setting:<br>• Field permit number<br>• Name of the institution or relevant body that granted permission	
**Data Availability**<br><br>Authors are required to make all data underlying the findings described fully available, without restriction, and from the time of publication. PLOS allows rare exceptions to address legal and ethical concerns. See the PLOS Data Policy and FAQ for detailed information.	No - some restrictions will apply

Powered by Editorial Manager® and ProduXion Manager® from Aries Systems Corporation

A Data Availability Statement describing where the data can be found is required at submission. Your answers to this question constitute the Data Availability Statement and **will be published in the article**, if accepted.<br><br>**Important:** Stating 'data available on request from the author' is not sufficient. If your data are only available upon request, select 'No' for the first question and explain your exceptional situation in the text box.<br><br>Do the authors confirm that all data underlying the findings described in their manuscript are fully available without restriction?	
**Describe where the data may be found in full sentences. If you are copying our sample text, replace any instances of XXX with the appropriate details.**<br><br>• If the data are **held or will be held in a public repository**, include URLs, accession numbers or DOIs. If this information will only be available after acceptance, indicate this by ticking the box below. For example: *All XXX files are available from the XXX database (accession number(s) XXX, XXX.*).<br>• If the data are all contained **within the manuscript and/or Supporting Information files**, enter the following: *All relevant data are within the manuscript and its Supporting Information files.*<br>• If neither of these applies but you are able to provide **details of access elsewhere**, with or without limitations, please do so. For example:<br><br>*Data cannot be shared publicly because of [XXX]. Data are available from the XXX Institutional Data Access / Ethics Committee (contact via XXX) for researchers who meet the criteria for access to confidential data.*<br><br>*The data underlying the results presented in the study are available from (include the name of the third party*	The secondary data used in the quantitative analysis has a third party ownership and can be access from MEASURE DHS at https://dhsprogram.com/data/).<br>However, the qualitative primary data is available and can be access from the first author.

Powered by Editorial Manager® and ProduXion Manager® from Aries Systems Corporation

and contact information or URL).<br>• This text is appropriate if the data are owned by a third party and authors do not have permission to share the data.<br><br>* typeset	
Additional data availability information:	Tick here if the URLs/accession numbers/DOIs will be available only after acceptance of the manuscript for publication so that we can ensure their inclusion before publication.

Manuscript Click here to access/download;Manuscript;Manuscript.docx

Evaluating the impact of maternal health care policy on stillbirth and perinatal mortality in Ghana; a mix method approach using two rounds of Ghana demographic and health survey data sets and qualitative design technique.

John Azaare[1,2], Patricia Akweongo[2], Genevieve Cecilia Aryeteey[2], Duah Dwomor[3]

[1]Department of Health Services, Policy Planning, Management and Economic, School of Public Health, University for Development Studies, Tamale, Ghana.

[2]Department of Health Policy, Planning and Management, School of Public Health, University of Ghana, Legon, Accra, Ghana.

[3]Department of Biostatistics, School Public Health, University of Ghana, Legon, Accra, Ghana.

Correspondent Email: *jazaarejn@uds.edu.gh (JA)*

Abstract

Background: Stillbirth and perinatal mortality indices appear to be declining at a slow pace in Ghana despite the 'free' maternal health care policy intervention since 2008, hence, raising concerns as to whether Ghana is on course to achieving the World Health Organization target of 12 per 1000 live births by the year 2030.

Purpose: In this study, we compared stillbirth and perinatal mortality outcomes between 2008 and 2014 using Ghana demographic and health survey data set since the 'free' policy inception. We further explored any contextual factors underpinning the policy operation to find explanation to the current situation relative to stillbirth and perinatal mortality.

Methods: The study adopted a mix method approach, first using two rounds of Ghana Demographic and Health Survey data sets, 2008 and 2014 as baseline and end line respectively. We constructed outcome variables of stillbirth and perinatal mortality from the under 5 mortality variables of two rounds of GDHS (n=487), and then estimated the overall rates and proportion of stillbirth and perinatal mortality for the two rounds. We then analysed for association using Poisson and multiple logistics regressions and also with negative binomial regression models to test for sensitivity, while adjusting for; maternal age, parity, area of residence, educational status, marital status, wealth index, and region as independent covariates, statistically significance, $p < 0.05$. We also conducted in-depth interviews for service providers and held focus group discussions with pregnant women to examine any contextual factors to draw conclusions and lessons for policy makers.

Results: The results show that stillbirth rate increased in 2014 by 2 per 1000 live births, while perinatal mortality rate declined within the same period by 4 per 1000 live births, compared 2008. However, both stillbirth and perinatal mortality increased in proportion between 2008 and 2014 by 14.4% and 10.8%, respectively. The analysis of association found that pregnant women were 1.64 times more likely to record stillbirth; aOR: 1.64; 95% [CI: 1.02, 2.65] and 2.04 times more likely to record perinatal mortality; aOR: 2.04; 95% [CI: 1.28, 3.25] among the 'free' maternal health care policy group, and these were statistically significant, $p < 0.041$; $p < 0.003$, respectively.

The qualitative study found that, routine medicines such as folic acid and multivitamine were in intermittently in short supply forcing private purchase by pregnant women to augment their routine requirement. Also, the study found that pregnant women in labour took local concoction as oxytocin ostensibly to fast track the labour process and inadvertently causing complications of uterine rapture increasing the likelihood of stillbirths.

Conclusion: Even though perinatal mortality rate decline overall between 2008 and 2014, the proportion of stillbirth and perinatal death is still on the rise, thus, the 'free' policy intervention has yet to translate to the desired decline in stillbirths since its inception in 2008. Shortage of medicine commodities, inadequate monitoring of labour process coupled with pregnant women intake of traditional herbs are probably some explanations to the current and perhaps influencing stillbirth outcomes.

Keywords: Impact, stillbirth, perinatal mortality, maternal health care, contextual factors.

Introduction

Stillbirth and perinatal mortality are a critical healthcare index that has yet to receive adequate research and policy attention, particularly in developing countries(1–3). Stillbirth in particular presents economic and social effect to families particularly, the affected mother often leading to social withdrawal, loneliness and depression, affecting the psychosocial wellbeing of affected persons (4).

Globally, 2.6 million pregnancies end in stillbirths for one reason or the other despite improvement in access to care (5–8). Presently, the data show that despite the introduction of access to care interventions in developing countries such as social health insurance, 98% of stillbirth are still accounted for by lower and middle income countries, particularly in south-east Asia and sub-Saharan Africa (9–11).

As at 2015, Nigeria recorded a stillbirth rate of 42 per 1000 live births, whereas developed countries like Japan and UK recorded an average of 2 per 1000 live births (12,13). About the same period Ghana recorded 28 per 1000 live births (14,15).

Generally, stillbirth is defined as a fetus with no sign of life prior to delivery or expulsion (16,17). Technically, the WHO defines stillbirth as intrauterine death after conception. In practice however, gestational age of 20 to 28 weeks or a birth weight of 350 to 1000g is usually required in health law to determine stillbirth (16).

Access to maternal health care is proven to reduce stillbirth, and thus, the government of Ghana declared the 'free' registration of pregnant women to its national health insurance scheme in 2008

aimed at bridging the access gap to care usually induced by poverty (18,19). While the 'free' policy targeted access to care in particular, its broader intention was envisaged at tackling not just maternal mortality, but also to achieve a reduction in neonatal mortality by the provision of comprehensive pregnancy and newborn care during the perinatal period with the hope of meeting the then millennium development goal 4 and 5 (now sustainable development goal 3) (20–25).

The 'free' policy initiative received financial pledge from the then UK government to the tune of £42.5 million as a seed money. The policy has since survived a decade in its implementation and covered over 3 million beneficiaries since its inception in 2008 (26,27).

In spite of the intervention, stillbirth rates in Ghana have yet to drop substantially to measure up to desired targets if Ghana were to achieve the World Health Organization (WHO) goal of 12 per 1000 live births by the year 2030 (28,29). So far, studies on the 'free' policy impact have centered mainly on maternal health care utilization across districts and also, cross-country comparison of infant mortality in the West African sub-region (19,30,31).

In contrast, the 'free' maternal health policy impact on stillbirth perinatal mortality has yet to receive adequate attention relative to implementation of the 'free' maternal health care policy. More so, while the data on skilled delivery improves following the 'free' maternal health care policy initiative, stillbirth still appears to be increasing even among facility deliveries (32,33).

Thus, in this paper, we analyse the outcomes of stillbirth and perinatal deaths among mothers in2008, when the policy had just started, compared to 2014 when the policy was fully rolled out. The study examined the 'free' policy association with the outcomes of interest to see the likelihood

stillbirth and perinatal death being recorded among mothers who benefited from the ‘free’ policy, compared to mothers who did not.

Even though tailored interventions have been shown to yield effect on reduction stillbirth, some other studies insist that reduced outcome in stillbirth are often dependent on local contest(34), hence, we finally explored the views of service providers and pregnant women as policy implementers and policy users respectively, to examine the context under which the ‘free’ policy thrives to aid policy review and brief.

Conceptual framework

We hypothesized that certain factors undermine the successful operationalization of the ‘free’ maternal health care policy in Ghana (Fig 1) and hinders the intention of the ‘free’ policy which was introduced to increase maternal health care utilization to bring about a decline in not just maternal mortality, but also stillbirth and perinatal mortality in the medium to long term.

The ‘free’ maternal health care policy is administered via the national health insurance scheme (NHIS) which in itself is bedeviled with funding constraints culminating in delays in payment of claims over the years (35,36). Consequently, it appears the existing challenges affect the effective management of accredited service provider facilities thereby threatening the credibility of the purchaser provider split concept (37).

On another level, the Ghanaian society presents itself as keeper of pregnant women with cultural demands that upset the effective implementation of the ‘free’ policy as well. These unintended

practices seem to derail efforts of health care professionals and policy makers and hence, seem to frustrate their efforts of achieving reduced mortality outcomes of new born care.

Studies have shown that maternal age, rural/urban area of residence, twin pregnancy, history of abortion, income level, education and marital status all play to moderate the outcome of stillbirth and perinatal mortality (38–40). In this current study, we ask, to what extend does the maternal health care policy intervention affect outcome of stillbirth and perinatal mortality against the background of the policy implementation bottleneck while accounting for the moderating factors, thus, the focus of the paper.

Fig 1. Conceptual framework: multifaceted factors moderating stillbirth and perinatal mortality

Contextual Definition

Stillbirth: In this study, we defined stillbirth to refer to born dead or dying within 24 hours after birth. This definition was critical to widen the scope of newborn death within hours after delivery

Perinatal mortality: We defied Perinatal Mortality to include all newborn deaths recorded within 6 days after birth.

'Free' maternal health care policy: We defined the Maternal Health Care Policy in this study to refer to pregnant women who registered with the national health insurance scheme (NHIS).

Materials and methods

Study design

Ghana implements the ‘free’ maternal health care policy via the National Health Insurance Scheme (NHIS), thus, we defined the ‘free’ maternal health care policy (FMHCP) to refer to pregnant women registrants of the NHIS. Giving the policy was started in 2008, we used Ghana Demographic and Health Survey (DHS) data set of 2008 as baseline while another round, 5 years post 2008 DHS data set (2014 GDHS) was used as our end line data sets. The two rounds (GDHS 2008/2014) were merged and registrants of the NHIS generated as beneficiaries of the ‘FMHCP’ according to our definition and compared to non-registrants of the NHIS (no-FMHCP).

We then selected 5 hospitals and 5 health centres from two regions of Ghana based on the current indices of stillbirth and perinatal mortality in the two regions, and conducted in-depth interviews for the health service providers and focus group discussions for the pregnant women users of the ‘free; maternal health care policy for a contextual understanding of the ‘free’ policy implementation.

Study setting

The quantitative analysis used Ghana Demography and Health Survey data sets, nationally representative, as data for the analysis. However, three districts each were considered from two regions of Ghana to explore the context within which the ‘free’ maternal health care is implemented relative to stillbirth and perinatal mortality. Both regions were selected from northern Ghana; the Upper East region and the Northern region (Fig 2).

The Upper East Region is one of the poorest regions of Ghana. It is located at the north-eastern corner of Ghana between longitude 0^0 and 1^0 West and latitude 10^0 30' N and 11^0 N. The region has a total land area of about 8,842sq km with Bolgatanga as its capital and an estimated population of a little over 1million (41,42). As at 2013, the region had a national health insurance enrollment rate of 6.3% of its total population with a considerably good number of midwives compared to other regions of Ghana (43). Three districts were selected from the UER for the primary data collection.

On the other hand, the Northern region is shared boundary to the Upper East Region, prior to 2018. As 2015, literacy rate was poor the Northern, less than 50% (44). The Northern region shares boundaries with the Republic of Togo to the East and the Savana Region to the south and the North-East region to the north. The region has a land mass stretching about 70,765.2km^2 with an estimated population of about 1.8m representing 9.6% of the total Ghanaian population. Three districts were also considered for the qualitative data from the Northern region.

Fig 2. Map of selected study areas of Upper East and Northern regions.

Study participants and sampling

Quantitative

The outcome variables; stillbirth and perinatal mortality were then generated from the under 5 mortality variables by constructing binary outcome of 1 and 0, thus, 1 referring to all mortalities within 6 days after birth as perinatal mortality and 0 referring to all other under 5 mortalities (day 7 and beyond) as no perinatal morality. Similarly, a binary outcome of 1 was constructed for

stillbirth, referring to born dead or dying within zero (24hours) and ‘0’ for all other under 5 mortalities (referring to no stillbirth).

Qualitative

Medical doctors, midwives and pregnant women were recruited from selected health care facilities across two region of Ghana for in-depth interviews (IDIs) and focus group discussions (FGDs). The regions were divided into three zones; from which one district each was selected using simple random sampling. A hospital each and two health centers per zone, were further selected as the final study sites from which study the participants were recruited purposively from the labour sections and the antenatal care units for the study. Pregnant women attending antenatal clinics in the selected facilities were also recruited conveniently for the focus group discussions. The use of multiple sources of data (service provider and pregnant women) was to explore the idea of multiple realities (45).

Data collection and analysis

Tools and pretesting

Interviews guides were developed and pre-tested among midwifery staff in a hospital which was not part of the selected study sites. We then revised the tools based on our observations. The Graduate Studies Committee of the University Ghana School of Public Health then approved of the tools and these were attached to the study protocol and received approval from Ghana Health Service Ethics Review Committee (letter attached) as tools for the in-depth interviews (IDIs) and focus group discussions (FGDs).

In-depth interviews were one-on-one for doctors and midwives using open ended interview questions. Each session lasted for about 1 hour and was audio taped. The focus groups ranged from 5 to 7 for the pregnant women participants who were attending antenatal care clinic at the selected health care facilities. We used fictional cases mainly at the FGDs to stimulate participation and aid discussion.

Inclusion criteria

The secondary data sets were those of the women data sets as contained in the 2008/2014 Ghana Demographic and Health Survey. Women respondents were between the ages of 15-49 years for both rounds. On the other hand, only doctors and midwives with at least 3 years working experience in the labour units and antenatal care sections were included for the in-depth interviews to be sure they have adequate experience working with the 'free' maternal health care policy.

Exclusion criteria

Pregnant women receiving treatment for a medical condition were excluded from the study. Also, pregnant women less than 16 years were considered as minors under the 1992 constitution of the Republic of Ghana, and also excluded from the focus group discussion.

Quality control and trustworthiness

Weighting was applied to the GDHS data sets to cater for clustering and stratification across rural and urban areas of the then ten regions of Ghana, and analysis run using Taylor linearization technique for reduced standard error. For the qualitative study, the choice of purposive sampling was deliberate to achieve trustworthiness through the acquisition of the right information from

service providers; doctors and midwives and pregnant women as policy users. To verify and triangulation data, we interviewed an expert Key Informant, a regional director of Ghana Health Service. This approach allowed us to validate the field data which was useful and catered for the idea of multiple sources of information as espoused by Creswell.

Statistical analysis

First, we estimated the overall ratios of stillbirth and perinatal mortality for the two rounds and also, the prevalence of stillbirth and perinatal mortality between the baseline and end line (2008 and 2014, respectively) to compare the outcomes, pre and post the 'free' policy intervention. We then identified confounding variables (Table 2) using Rao-Scot chi square which were included in Poisson and multiple logistic regression models as covariates for the analysis of association.

Only variables which were statistically significant ($p < 0.05$) in association with the outcome variables; stillbirth and perinatal mortality were included in the regression models. However, variable such as twin pregnancy ($p = 0.4114$), wealth index ($p = 0.2408$), and respondent region ($p = 0.7535$) were also included as confounding based on Mosley and Chen framework for child survival as modified in our conceptual framework even though these did not show statistically significant association with the outcome variables (46,47).

Thus, maternal age, area of residence, employment status, history of abortion, twin pregnancy, caesarean section, marital status, educational status, wealth quintile and region were adjusted for as confounding. Weighted was applied by the selection of variable 005 (sample weighting), variable 021 (clustering) and variable 022 (sample stratum) from the complex DHS data sets using STATA 15 to cater for clustering and stratification. Thereafter, all Stata commands prefix *'svy'* to

ensure that the analysis applied weighting across DHS data. We then checked for sensitivity and over dispersion using negative binomial regression model. The results of analysis of association are reported in adjusted odds ratio and adjusted prevalent ratio.

Qualitative data analysis

Interviews and group discussions were transcribed verbatim into Microsoft office, double-checked for accuracy, and imported into INVIVO 10 for word cloud view. Subsequent analysis was largely manual through multiple reading where data were grouped into similar and dissimilar statements with annotation while bringing significant statements under constructed themes.

Significant statements were coded and categorized under the themes, reviewing each theme carefully for relevance. Statements that did not align themselves to the study objective was thoroughly examined for relevance or excluded altogether. Constructed themes were based on common phrases and similar statement approach and relevant statements quoted verbatim in reporting the results to convey participants' impression and aid explanatory power.

Ethical consideration

This study is part of the PhD thesis research of the first author and received ethical clearance from the Ghana Health Service Ethical Review Board reg. no. GHS-ERC: 002/04/19. The secondary data was obtained from MEASURE DHS after completing an online application process, and thus, follows a third party user guidelines as stated in the letter from Measure DHS, USA. All the study participants for the primary data collection consented to being part of this study and signed or thumb printed consent form to participate.

In-depth interviews were conducted in private rooms, whereas focus group discussions were held in open space under chalets for aeration and as part of COVID-19 protocol. Assurance of anonymity and non-disclosure was also offered to the pregnant women. The study protocol was also peer reviewed by four double blinded external reviewers and published by Biomed Central Reproductive Health Journal (27).

Results

Quantitative findings

Weighted Distribution of Maternal and Population Characteristics between 2008 and 2014

The weighted distribution of maternal characteristics in Table 1 shows that women respondents were majorly between the ages of 25 and 29 years for both the 2008 and the 2014 groups, 824 (27.9%) and 1494 (24.9%), respectively. The differences in comparison between the group ages are also statistically significant, p= 0.0044. Respondents from rural areas were slightly higher compared to those from urban areas, 1992 (62.1%) for 2008 and 3540 (55.0%) for 2014, compared to the respondents from the urban areas. Majority of the respondents were employed for both years, 2609 (87.5%) and 4663 (79.4%) for 2008 and 2014 respectively, compared to those without employment.

Pregnancy histories were mainly singleton, 2860 (95.6%) and 5597 (94.8%) for 2008 and 2014, respectively, with Caesarean section (CS) delivery of 182 (6.9%) in the 2008 and increased slightly to 616 (12.8%) in 2014. The difference is statistically significant, $p < 0.0001$. on marital status, 2214 (70.8%) were married in 2008 compared to 3918 (63.3%) in 2014.

Most respondents attained secondary level of education, with 1073 (40.1%) and 2409 (48.1%) reporting this level of education respectively for 2008 and 2014. Most women respondents were in the poorest wealth quintile, 973 (25.6%) in 2008 and 1886 (22.2%) in 2014, followed by the poorer wealth quintile, 656 (22.0%) in 2008 and 1304 (21.0%) in 2014. Of the total of 2992 respondents in 2008, 439 (18.7%) were from the Ashanti region followed by 479 (15.7%) from the Northern region.

Table 1. Weighted maternal and population characteristics adjusted for as confounding

Variable	Obs.	2008 (%)	2014 (%)	Pearson Design-based F test (p-value)
Maternal Age				0.0044*
15-19	329	119 (4.0)	207 (3.6)	
20-24	1592	583 (19.5)	1009 (17.0)	
25-29	2318	824 (27.9)	1494 (24.9)	
30-34	2012	603 (20.3)	1409 (24.9)	
35-39	1581	520 (17.2)	1061 (18.4)	
40-44	771	242 (7.7)	529 (8.6)	
45-49	276	101 (3.3)	175 (2.5)	
Total	8876	2992 (100)	5884 (100)	
Area of Residence				0.0410*
Urban	3344	1000 (37.9)	2344 (45.0)	
Rural	5582	1992 (62.1)	3540 (55.0)	
Total	8876	2992 (100)	5884 (100)	
Employment Status				0.0001*
Unemployed	1581	364 (12.5)	1217 (20.6)	
Employed	7272	2609 (87.5)	4663 (79.4)	
Total	8853	2973 (100)	5880 (100)	
History of Abortion				
No abortion	7138	2547 (84.1)	4591 (74.4)	
Abortion	1732	439 (15.9)	1293 (25.6)	
Total	8870	2986 (100)	5884 (100)	
Twin Pregnancy				0.4114
Singleton	8457	2860 (95.6)	5597 (94.8)	
1st twin	209	66 (2.2)	143 (2.6)	
2nd twin	209	66 (2.2)	143 (2.6)	

Total	8876	2992 (100)	5884 (100)	
**Caesarean Section**				0.0001*
Vaginal delivery	8075	2807 (93.1)	5268 (87.2)	
Caesarean delivery	798	182 (6.9)	616 (12.8)	
Total	8876	2992 (100)	5884 (100)	
**Marital Status**				0.0001*
Never married	534	130 (4.9)	404 (7.6)	
Married	6163	2214 (70.8)	3918 (63.3)	
liv. together	1687	498 (18.6)	1189 (22.4)	
Widow	127	29 (1.0)	98 (1.3)	
Divorced	125	47 (1.1)	78 (1.3)	
Not living together	271	74 (2.7)	197 (4.0)	
Total	8876	2992 (100)	5884 (100)	
**Education Status**				0.0003*
No education	3174	1132 (32.7)	2042 (27.4)	
Primary education	1931	722 (24.8)	1209 (20.0)	
Secondary education	3482	1073 (40.1)	2409 (48.1)	
Tertiary education	287	65 (2.4)	224 (4.5)	
Total	8876	2992 (100)	5884 (100)	
**Wealth Quintile**				0.2408
Poorest	2859	973 (25.6)	1886 (22.2)	
Poorer	1960	656 (22.0)	1304 (21.0)	
Middle	1587	504 (18.9)	1083 (19.6)	
Richer	1385	502 (19.3)	883 (18.8)	
Richest	1085	357 (14.3)	728 (18.4)	
Total	8876	2992 (100)	5884 (100)	
**Respondent Region**				0.7535
Western	852	270 (9.3)	582 (10.0)	
Central	830	227 (10.0)	603 (10.9)	
G. Accra	739	279 (11.9)	460 (15.5)	
Volta	726	245 (8.4)	481 (7.7)	
Eastern	806	261 (8.8)	545 (9.4)	

Ashanti	1038	439 (18.7)	599 (18.7)	
Brong-Ahafo	919	266 (9.3)	653 (8.7)	
Northern	1381	479 (15.7)	902 (12.5)	
Upper East	778	227 (5.0)	551 (3.9)	
Upper West	807	299 (2.8)	508 (2.7)	
Total	8876	2992 (100)	5844 (100)	

**significance level: *p< 0.05*

Prevalence of Stillbirth and Perinatal Mortality Between 2008 and 2014

As shows in Table 2, a total of 174 stillbirths were recorded between the two rounds of Ghana Demographic and Health Survey. Of this number, 55 (28.7%) were recorded in 2008, compared to 119 (43.1%) in 2014, thus showing an increase of 14.4%. Also, a total of 243 perinatal mortalities were recorded between 2008 and 2014 perinatal mortality increased, 88 (45.6%) in 2008 compared to 155 (56.4%) in the 2014, representing an increase of 10.8% but at a decreasing rate comparing to the stillbirth within the same period.

Table 2. Proportion of stillbirths and Perinatal Mortality in 2008 and 2014

Variable	Obs.	2008 (%)	2014 (%)	Pearson Design-based F test (p-value)
**Stillbirth**				0.0067*
Not stillborn	313	143 (71.3)	170 (56.9)	
Stillborn	174	55 (28.7)	119 (43.1)	
Total	487	198 (100)	289 (100)	
**Perinatal Mortality**				0.0344*
Not recorded	244	110 (54.4)	134 (43.6)	
Perinatal Death	243	88 (45.6)	155 (56.4)	
Total	487	198 (100)	289 (100)	

Stillbirth and Perinatal Mortality Rate between 2008 and 2014

The overall rate of stillbirth and perinatal mortality (Table 3) between the two rounds for the 2008 and 2014 period shows that stillbirth rate increased by 2% in 2014 compared to 2008 moving from 19 per 1000 live births to 21 per 1000 live births in 2014. However, for perinatal mortality the estimated figures showed a slide improvement declining by 4 per 1000 live births moving from 31 per 1000 live births in 2008 to 27 per 1000 live births in 2014.

Table 3. Estimated Stillbirth and Perinatal Mortality Rates for 2008 and 2014

Variable	Rate	2008	2014
Stillbirth	Per 1000 live births	19	21
Perinatal Mortality	Per 1000 live births	31	27

Calculations of Rates

Stillbirth Rate

$$\frac{55}{55+2794}\text{X}1000$$

= 0.0193

= 19 per 1000 live births (2008)

$$\frac{119}{119+5595}\text{X}1000$$

= 0.0208

= 21 per 1000 live births (2014)

Perinatal Mortality

$$\frac{88}{55+2794}\text{X}1000$$

= 0.0308

= 31 per 1000 live births (2008)

Perinatal Mortality Rate

$$\frac{155}{119+5595}\text{X}1000$$

= 0.0271

= 27 per 1000 live births (2014)

Association between the 'free' maternal health care policy and risk of stillbirth

On risk of stillbirth, pregnancies in the 'Free' Maternal Health Policy (FMHCP) group were 1.64 times more likely to result in stillbirth compared to the no FMHCP group; aOR: 1.64; 95% CI: 1.02 to 2.65; p= 0.041. This is shown in Table 4 and are similar for Poisson and negative binomial regressions models, aPR: 1.34; 95% CI: 1.00 to 1.79; p= 0.045 respectively. Unexpectedly, women with secondary level of education were also more likely to register stillbirth compared to women with no formal education, and this was statistically significant, aOR: 2.02; 95% CI: 1.06 to 3.84; p= 0.030.

Table 4. Association Between the Free Maternal Health Care Policy and Risk of Stillbirth using Poisson, Binary Logistics and Negative Binomial Regressions with Linearized Standard Error

Stillbirth	Logistic regression with Linearized standard error			Poisson regression with linearized standard error			Negative binomial regression with robust std. error		
	aOR	(CI: 95%)	P-Value	aPR	(CI: 95%)	P-value	aPR	(CI: 95%)	P-value
**Policy intervention**									
No_FMHCP	1			1			1		
FMHCP	1.64	(1.02-2.65)	0.041*	1.34	(1.00-1.79)	0.045*	1.34	(1.00-1.79)	0.045*
**Maternal age**	1.05	(1.01-1.08)	0.007*	1.02	(1.00-1.05)	0.006*	1.02	(1.00-1.05)	0.006*
**Twin pregnancy**									
Singleton	1			1			1		
1st set of twins	1.39	(0.66-2.90)	0.375	1.22	(0.83-1.77)	0.296	1.22	(0.83-1.77)	0.296
2nd set of twins	1.24	(0.43-3.56)	0.680	1.15	(0.66-2.00)	0.609	1.15	(0.66-2.00)	0.609
3rd set of twins	-	-	-	1.99	(1.20-3.20)	0.008*	1.99	(1.20-3.20)	0.008*
**Caesarean section**									
No	1			1			1		
Yes	2.10	(0.94-4.67)	0.067	1.36	(0.96-1.94)	0.084	1.36	(0.96-1.94)	0.084
**Abortion history**									
No	1			1			1		
Yes	1.04	(0.59-1.84)	0.875	1.02	(0.75-1.37)	0.906	1.02	(0.75-1.37)	0.906
**Area of residence**									
Urban	1			1			1		
Rural	1.82	(0.95-3.48)	0.067	1.37	(0.97-1.95)	0.072	1.37	(0.97-1.95)	0.072
**Educational status**									
No education	1			1			1		
Primary	1.26	(0.66-2.40)	0.472	1.14	(0.77-1.68)	0.505	1.14	(0.77-1.68)	0.505
Secondary	2.02	(1.06-3.84)	0.030*	1.47	(1.01-2.13)	0.041*	1.47	(1.01-2.13)	0.041*
Tertiary	0.25	(0.03-1.74)	0.163	0.42	(0.10-1.71)	0.229	0.42	(0.10-1.71)	0.229
**Wealth index**									
Poorest	1			1			1		
Poorer	0.95	(0.48-1.88)	0.895	0.99	(0.65-1.50)	0.966	0.99	(0.65-1.50)	0.966
Middle	1.38	(0.65-2.90)	0.394	1.22	(0.79-1.90)	0.362	1.22	(0.79-1.90)	0.362

Richer	1.26	(0.87-5.84)	0.090	1.52	(0.92-2.53)	0.101	1.52	(0.92-2.53)	0.101
Richest	1.64	(0.53-5.04)	0.383	1.33	(0.70-2.54)	0.378	1.33	(0.70-2.54)	0.378
**Region**									
Western	1			1			1		
Central	0.74	(0.31-1.78)	0.508	0.81	(0.48-1.36)	0.440	0.81	(0.48-1.36)	0.440
G. Accra	1.59	(0.50-5.04)	0.429	1.29	(0.69-2.41)	0.407	1.29	(0.69-2.41)	0.407
Volta	2.54	(0.74-8.64)	0.134	1.55	(0.86-2.79)	0.141	1.55	(0.86-2.79)	0.141
Eastern	1.42	(0.58-3.48)	0.439	1.23	(0.73-2.05)	0.436	1.23	(0.73-2.05)	0.436
Ashanti	1.15	(0.46-2.88)	0.761	1.08	(0.64-1.85)	0.749	1.08	(0.64-1.85)	0.749
Brong-Ahafo	0.92	(0.33-2.58)	0.881	0.93	(0.50-1.75)	0.837	0.93	(0.50-1.75)	0.837
Northern	1.02	(0.41-2.54)	0.959	0.96	(0.54-1.69)	0.892	0.96	(0.54-1.69)	0.892
Upper East	1.08	(0.33-3.55)	0.888	1.03	(0.47-2.22)	0.940	1.03	(0.47-2.22)	0.940
Upper West	1.15	(0.45-2.95)	0.761	1.08	(0.61-1.89)	0.785	1.08	(0.61-1.89)	0.785

Notation: 1 *– reference;* ***aOR*** *– adjusted Odd Ratio****; aPR*** *– adjusted Prevalence Ratio; * p<0.05.*

Association between the 'free' maternal health care policy and risk of perinatal mortality

From Table 5, the results also show that newborns were 2.04 times more likely to die within 6 days of life in the FMHCP group compared to their counterparts in the no FMHCP group, aOR: 2.04; 95% CI: 1.28-3.25; p= 0.003. This also compares similarly for Poisson and Negative binomial regressions, aPR: 1.34 and these were statistically significant, p=0.006, respectively.

Women with secondary level of education were also more likely to register perinatal mortality compared to women with no formal education, aOR: 1.89; 95% CI: 0.98 to 3.63. However, this was not statistically significant, p= 0.056. Rather, women with history of abortion were more likely to record perinatal mortality compared to women with no history of abortion, and this was statically significant, aOR: 1.91; 95% CI: 1.07 to 3.41; p= 0.028.

Table 5. Association Between the Free Maternal Health Care Policy and Risk of Perinatal Mortality using Poisson, Binary Logistics and Negative Binomial Regressions with Linearized Standard Error

Perinatal Mortality	Poisson regression with Linearized std. error			Binary logistics regression with linearized std. error			Negative binomial regression with linearized std. error		
	aOR	(CI: 95%)	P-Value	aPR	(CI: 95%)	P-Value	aPR	(CI: 95%)	P-Value
**The Policy**									
No_FMHCP	1			1			1		
FMHCP	2.04	(1.28-3.25)	0.003*	1.35	(1.08-1.66)	0.006*	1.35	(1.08-1.66)	0.006*
Maternal age	1.03	(0.99-1.06)	0.100	1.01	(0.99-1.03)	0.101	1.01	(0.99-1.03)	0.101
**Twin pregnancy**									
Singleton	1			1			1		
1st set of twins	1.37	(0.67-2.80)	0.377	1.14	(0.87-1.49)	0.331	1.14	(0.87-1.49)	0.331
2nd set of twins	1.82	(0.70-4.72)	0.218	1.24	(0.91-1.69)	0.166	1.24	(0.91-1.69)	0.166
3rd set of twins	1	-	-	1.43	(1.03-1.98)	0.033*	1.43	(1.03-1.98)	0.033*
**Cesarean section**									
No	1			1			1		
Yes	2.02	(0.87-4.66)	0.100	1.25	(0.97-1.60)	0.075	1.25	(0.97-1.60)	0.075
**Abortion history**									
No	1			1			1		
Yes	1.91	(1.07-3.41)	0.028*	1.25	(1.02-1.53)	0.031*	1.25	(1.02-1.53)	0.031*
**Area of residence**									
Urban	1			1			1		
Rural	1.28	(0.70-2.32)	0.407	1.11	(0.87-1.42)	0.366	1.11	(0.87-1.42)	0.366
**Education**									
No education	1			1			1		
Primary	0.93	(0.49-1.74)	0.822	1.97	(0.71-1.31)	0.863	1.97	(0.71-1.31)	0.863
Secondary	1.89	(0.98-3.63)	0.056	1.30	(0.97-1.72)	0.071	1.30	(0.97-1.72)	0.071
Tertiary	0.25	(0.04-1.44)	0.124	0.52	(0.19-1.40)	0.196	0.52	(0.19-1.40)	0.196
**Wealth index**									
Poorest	1			1			1		

Poorer	0.56	(0.26-1.23)	0.153	0.80	(0.56-1.13)	0.207	0.80	(0.56-1.13)	0.207
Middle	0.84	(0.35-1.99)	0.694	0.93	(0.66-1.31)	0.690	0.93	(0.66-1.31)	0.690
Richer	0.87	(0.32-2.35)	0.789	0.95	(0.65-1.39)	0.818	0.95	(0.65-1.39)	0.818
Richest	0.76	(0.24-2.40)	0.641	0.90	(0.58-1.38)	0.639	0.90	(0.58-1.38)	0.639
**Region**									
Western	1			1			1		
Central	0.63	(0.24-1.61)	0.340	0.79	(0.52-1.18)	0.258	0.79	(0.52-1.18)	0.258
G. Accra	1.73	(0.54-5.56)	0.353	1.20	(0.77-1.86)	0.412	1.20	(0.77-1.86)	0.412
Volta	2.79	(0.77-10.0)	0.116	1.32	(0.89-1.97)	0.159	1.32	(0.89-1.97)	0.159
Eastern	1.36	(0.51-3.62)	0.526	1.11	(0.76-1.62)	0.577	1.11	(0.76-1.62)	0.577
Ashanti	1.03	(0.38-2.78)	0.949	1.00	(0.67-1.48)	0.986	1.00	(0.67-1.48)	0.986
Brong-Ahafo	0.87	(0.30-2.50)	0.809	0.93	(0.60-1.43)	0.754	0.93	(0.60-1.43)	0.754
Northern	0.59	(0.21-1.64)	0.313	0.74	(0.47-1.17)	0.199	0.74	(0.47-1.17)	0.199
Upper East	0.45	(0.12-1.59)	0.217	0.64	(0.52-1.27)	0.200	0.64	(0.52-1.27)	0.200
Upper West	0.65	(0.23-1.85)	0.421	0.81	(0.61-1.89)	0.375	0.81	(0.61-1.89)	0.375

Notation: 1 *– reference;* ***aOR*** *– adjusted Odds Ratio****; aPR*** *– adjusted Prevalence Ratio; *p < 0.05.*

Qualitative findings

Study participants

A total of 67 primary participants (Table 6) granted in-depth interview and focus group discussions. Study participants were mainly females, 61 (91%). Of the total qualitative participants (Tables 6 and 7), 23 (34%) were health service providers comprising of midwives 18 (78%), and medical doctors/directors 5 (22%). The rest of the qualitative participants were pregnant women, 44 (66%) who participated in the focus group discussion. All pregnant women participants were active card bearing members of the National Health Insurance Scheme (proxy to the 'free' maternal health care policy). Majorly, pregnant women participants were married, 43 (98%), while 38 (86%) were employed. The majority of pregnant women respondents had given birth previously, 41 (90.1%). Only 4 (8.9%) pregnant women participants were having children for the first time. Table 6 presents details composition of the qualitative study participants.

Table 6. Distribution of Qualitative Study Participants

Method	Target	Size	Average Duration
In-depth interview	Doctors/directors	5	5 hour
In-depth interview	Midwives	18	12 hour
Group discussion	Pregnant women	44	6 hours
**Total**		**67**	**23 hours+**

Table 7. Characteristics of Qualitative Study Participants

Characteristics of study participants	Frequency (%)
**Category of study Participant**	
Service Providers	23 (34%)
Pregnant women	44 (66%)
Total	67 (100%)
**Gender of study participants**	
Male	6 (9%)
Female	61 (91%)
Total	67 (100%)
**Profession (service providers)**	
Midwife	18 (78%)
Doctor	5 (22%)
Total	23 (100%)
**Years in service (service providers)**	
>10years	6 (26%)
6-10yeas	12 (52%)
3-5years	5 (22%)
Total	23 (100%)
**NHIS status (pregnant women)**	
Registrant	44 (100%)
Non-registrants	0 (0%)
Total	44 (100%)
**Marital Status (pregnant women)**	
Married	43 (98%)
Not married	2 (2%)
Total	44 (100%)
**Employment Status (pregnant women)**	
Employed	38 (86%)
Unemployed	6 (14%)
Total	44 (100%)
**Parity (pregnant women)**	
Prime parity	4 (9%)
Multiparty	40 (81%)
Total	44 (100%)

Common Themes

In all, four themes were constructed, thus; increasing numbers of stillbirth, poor use of delivery partograph for labour monitoring, use of local herbs for ‘rapid’ uterine contraction, and intermittent shortage of medicine commodities.

Increasing Numbers of Stillbirth

Service providers acknowledges that stillbirth was on the rise, but attributes this to the increasing numbers of attendance and improved record keeping. Service providers are of the view that the increase in utilization puts pressure on the available resources and coupled with improved data capture, stillbirth figures appear to be worse of. They explain, thus;

“…In the region, when you look at the picture, despite the so many interventions, one will say SBs are still high. But when you look at it critically, it is the reporting which is also going up, so it makes you think that the policy is not helping…” ***(Doctor 1, IDI, UER)***

“…We have a high rate. This time we are getting mothers who are coming with Intra-Uterine Fetal Death (IUFD). This year in particular we had 15 for the first 6months. When you compare, I will say because we are taking records, that is why the numbers are high…previously there was no documentation…" ***(Midwife 1, IDI, Zebilla District)***

To the service providers, macerated stillbirths were being reported more, which suggest that babies die in utiro before they arrive at the facility. Service providers were also of the view that pregnant women reported late to health care facilities, sometimes to avoid procedures they appear uncomfortable with.

"…Actually, just this half-year, stillbirth numbers weren't encouraging. It was bad. We had 22, but 13 were macerated. Then we had 9 fresh stillbirths. The numbers are going up [increased]…" ***(Midwife 1, IDI, Bongo District)***

"…A few cases also dodge the hospital may they have two previous CS and knows that if they come to the hospital, there will be CS, so they avoid the hospital, when there are complications, then they quickly come..." ***(Doctor 1, IDI, UER)***

"…and when they come we manage them at postnatal care…. they say they didn't know that labour had started, some say they don't want the examination. One woman was frank, she said when they come, they put fingers on her vagina and that one she doesn't like it…" ***(Doctor 2, IDI, UER).***

Poor use of delivery partograph for labour monitoring

It merged that midwifery labour management was inadequate. Labour progress are said to be poorly monitored as delivery partograph, a tool recommended by the World Health Organization, is commonly inadequately administered, particularly in the Upper East region. A senior medical officer and director of the Regional Health Services**;**

"…Even though we use partograph to monitor labour we realize a substantial number are not monitored. Using partograph to monitor will tell you the condition of that baby. So that if you realize that the baby has difficulties, that baby can be delivered [via caesarean section]." ***(RDHS, KII, UER)***

"…Yes! A significant proportion are not monitored. Those women who are eligible, it should be 100%…" ***(RDHS, KII, UER)***

"As for partograph dier! It is 0 out 100 in…hospital [a particular hospital]. We went for monitoring on behalf of UNICEF and what we saw was not good all at" ***(Midwife 2, Tamale West)***

Service providers also observed a culture of little or no attention to stillbirth issues. Public or media lack of attention on stillbirth was cited as a principal reason compared to maternal mortality. On the other hand, pregnant women midwives' snobbish attitude to them during labour, as a contributory factor to the rising stillbirths. The following quotes explains further;

"We don't pay attention to stillbirth the way we do for maternal deaths. One mother will die and the whole hospital will here. I don't even know the stillbirths in the labour ward. They don't tell me…unless we are reporting. But when there is maternal mortality, eeeiii!" ***(Doctor3, IDI, UER)***

The views expressed by the medical doctors appear to be in consonance with a rather unethical practice exhibited by midwives which caught the attention of the pregnant women. The focus group discussion brought out that midwives hardly respond to the urgent calls of pregnant women in labour. Even though the reasons for their non-response was beyond the scope of this study, the descriptions during the focus group discussion suggest an underlying challenge that explains the poor outcomes of stillbirths. The pregnant women participants had these to say;

"…Sometimes you can be crying and they won't mind you. One time I was suffering and the midwives didn't bother to check on me. I said my baby is coming….by the time they came my baby was gone. They don't care about our babies…" ***(PW4, FG3, Bongo District)***

“…they [midwives] don’t pay attention to our babies. One woman nearly gave birth on the bench. She was calling the midwives…the baby is coming; the baby is coming. Oh! I felt very sad” ***(PW2, FG1, Zebilla Distric)***

Unsurprisingly, midwives noted that pregnant women demonstrated laid back attitude towards the care of their unborn babies, sometimes refusing surgery as may be required and often times failing to give inaccurate reproductive history which affect the care giving process and hence influence newborn outcomes. Charge midwives had these to say;

“… a woman came and the liquor was small, so the best we could do was CS. When we told them, they told us that if the water is not ok, can’t you fetch water and add. They went home…came back some few days later and the baby was dead…” ***(Midwife 2, IDI, Zebilla District)***

"…taking history is key... The woman misled the midwives concerning her parity. We started inducing, and she raptured, then, we asked a relative (her daughter) and she said her mother had 6 children and 1 died. Such a person should be induced…we were misled.” ***(Midwife 2, IDI, Bongo District)***

Use of local herbs for ‘rapid’ uterine contraction

Another interesting phenomenon that emerged from the IDIs was the use of local herbs for uterine contraction among pregnant women in labour. *‘Kaligutiem’* is a Dagbani (a Ghanaian language) term referring to herbal potion used by pregnant women to speed uterine contractions in labour, essentially acting as oxytocin. Unspecified doses of kaligutiem is given to some pregnant women from the study site hospital often by their mother in-laws and at the blind side of the health care professionals, to ‘aid’ the labour process.

The midwives account suggests that the potency of the locally produced oxytocin contributes to adverse events such as excessive contractions often leading to uterine rapture. Specific communities are notorious for the use of the local herb and mother in-laws are said to be the givers of this 'medicine' to quicken the labour process. Midwives and pregnant women give their account as follows;

"They also take, 'kaligutiem' [local oxytocin to aid uterine contraction] before coming to the hospital, and when they come the contractions will be too high…" ***(Midwife 2, IDI, Sagnarigu District)***

"Some also come with excessive contraction because of the 'kalgutiem', especially those coming from Dotoyille and Kunyevilla. The mother in-laws. They will give it to them and follow them to hospital as well" ***(Midwife 1, IDI, Sagnarigu District).***

Pregnant women admitted to knowing about *kaligutiem* during FGD as follows;

"We have heard about 'kuligutiem', but we don't use it. We don't know anybody who uses it. The midwives have complained and have advised us against it. ***(Pregnant Woman 3, FGD1, Sagnarigu).***

Intermittent shortage of medicine commodities

Another phenomenon sandwiching the 'free' policy implementation from both the service providers and pregnant women perspectives was frequent shortages of drug supplies for antenatal care and labour use at the health care facility level. Due to the facility level shortages,

pregnant women regularly asked to purchased medicines outside of the health facility set up to augment their required routine intake.

This was not only a problem to the pregnant women, but also frustrates the quality of processes of the health care professionals. Three medical doctors at post to the antenatal care units and delivery suits in the Upper East Region (UER) had these to say;

"…Our environment is not good. Personal hygiene is poor. Unlike other places where they think that labour is a sterile procedure, here, we routinely put all our clients on antibiotic cover, whether you're on episiotomy, assisted delivery, or not." ***(Doctor 1, IDI, UER)***

"…when you visit the facility and certain medication is not available, they are written for you in a prescription. So far as our facility is concern, if a medication is not available…we put it on a prescription for you to find a pharmacy shop to procure…" ***(Doctor 1, IDI, UER)***

"…The issue has to do with drugs. The challenge here is that most of the time the hospital runs out of stock. When they run out of stock, the patient must buy… because of the poverty level, most of them cannot afford the drugs…" ***(Doctor 2, IDI, UER)***

"…we use Antibiotic and pain killers for Cesarean Section. Then we also have hematinic. The better once, usually we want them to buy those outsides…. eenh! And IV fluids too. There are certain times we go virtually down, they have to buy virtually everything" ***(Doctor 3, IDI, UER)***

The midwifery staff equally bemoaned the difficulty in getting drugs at the facility level, particularly as some of these drugs are considered emergency requirement and its absence have direct consequences on newborn care and outcomes.

"…and after that, they pay for vitamin k, which we give to the child…that is when it is a normal delivery. When it is a Cesarean section, antibiotics like Cefuroxime, Amoxyclav, and Gentamycin are ordered by the doctor. If it is not there they go to buy…" ***(Midwife 2, KNNM, UER)***

"…When the dispensary does not have hematinic (iron III), you ask them to go and buy, it is a problem…. what about if she comes for ANC and you write for her and in the end she goes and not buy? She will come back with anaemia…" ***(Midwife 3, IDI, Bongo District)***

The pregnant women confirmed their predicaments during the focus group discussions as well;

"…Whenever we come we have been buying the drugs. Most of the time when we come they do write for us to go and buy the drugs. The yellow and the red drugs" ***(PW1, FG1, Bongo District)***.

"…the last time I delivered, my husband was made to buy water [intravenous fluids] for infusion…the is a drug store outside the hospital, that's where we bought it." ***(PW5, FG2, KNNM, UER)***

Discussion

Generally, the study results across the independent covariates improved over time between 2008 and 2014 showing statistically significant differences between the GDHS data of the two rounds, particularly in the weighted characteristics. Similarly, there is a corresponding increase in nominal values among the outcomes variables; stillbirth and perinatal mortality. Population growth is one possible explanation, however, the introduction of the 'free' maternal health care policy was also key to these improvements (48–50).

In other related studies, maternal health care utilization improved drastically following the introduction of the ‘free’ policy, hence showing some congruence with the current study (30,48,51). Also consistent with result of the analysis is the rising numbers of stillbirth among health facility delivery. The service providers disclosed this as yet a concern during the in-depth interview and focus group discussions as concern which was common despite the implementation of the ‘free’ policy.

The distribution of stillbirth between the two rounds was almost 70 percent accounted for by the 2014 with a statistically significant difference, $p < 0.0344$. Overall, we found that perinatal mortality rate declined in 2014 by 4 per 1000 live births, moving from 31 per 1000 live births to 27 per 1000 live births in 2008. However, stillbirth rate increased over the same period by 2 per 1000 live births in 2014 showing that while perinatal mortality was declining, stillbirth rate was rather rising. The service providers confirmed the fact that stillbirth was on the rise during the in-depth interviews, thus, lending credence to the quantitative results.

The findings show an inverse relationship between stillbirth and perinatal mortality outcomes within the same period which is rather surprising. Our attempt to explore the current situation qualitatively reveals that even though the ‘free’ policy may have opened access to maternal health care, this is against a background of shortage of medical consumable for expectant mothers, poor and inadequate monitoring of pregnancy and intermittent out-of-pocket expenditure on drugs, and diagnostics by pregnant women and their relatives.

Despite the overall improvement in outcome of perinatal mortality, perinatal mortality still increased proportionately between 2008 and 2014 by 10.8% moving from 45.6% of under-five mortality to 56.4% of under-five mortality within the DHS survey period.

The results compare intriguingly in proportion of stillbirth between 2008 and 2004 showing a much higher rise in proportions of stillbirth, 14.4% in 2014 compared to 2008 despite the existence the 'free' policy. The findings imply that both perinatal mortality and stillbirth did not exactly benefit from the 'free' policy intervention as envisaged, particularly in 2014 when the policy was in full implementation force.

The IDIs found that, first; increased utilization may have put pressure on the health care facilities and outstretch the already limited human and capital resources thereby affecting quality of care. Secondly, much more complicated cases which hitherto may not have reported to the hospital are more likely to end up in the health care facility, now that out of pocket payment have been minimized.

On the other hand, the slight improvement in perinatal mortality may be due to the increase access to care and immunization in 2014 compared to 2008. Perinatal mortality is technically an epidemiological concept that includes stillbirth and therefore, a reasonable expectation for us was that as one declines, the other should also be declining as well (16,38,52).

Service providers noted in the IDIs that medicines were intermittently in short supply as pregnant women are often told to purchase some for themselves from private chemical shops and this could perhaps affect late pregnancy outcomes. For example, folic Acid, and ferrous sulphate (iron

tablets) are routine drugs served at antenatal clinics as supplements to prevent anemia in pregnant women and these also aid in combating stillbirth (40,53).

In earlier studies, Mensah and others observed that 65% of pregnant women in rural Ghana take some form of antibiotics in addition to the routine medicines (54). With this, two possible scenarios could arise from the contextual factors. First, the risk of taking fake drug from the open market increased, and secondly, there is the likelihood of pregnant women not completing their required dose if they cannot afford the medicines from the market. Studies have shown that folic acid intake during pregnancy is associated with reduced stillbirth (55).

Another key finding from the qualitative analysis which may offer explanation to the rising stillbirth was the lack of ultra sound sonographers in most of the hospitals, particularly in the Upper East Region. All three hospitals studied in the Upper East region had no single sonographer to view and interprete ultrasound scan to aid the work of doctors and midwives. This led to a wide patronage of private ultra sound scans services to aid obstetric diagnosis.

Even though maternal health care utilization has increased, and perhaps a rise in record keeping, but this is against a background of diagnostic difficulties effectively relaying what Roos et. al., (2016) argues as contributory factors to undesirable outcomes of late pregnancy outcomes (6).

The ability of the service to accurately diagnose an obstetric condition is a functional quality issue and perhaps, hardly achieved in the current structure of services under the ‘free’ maternal health care policy (56,57).

Interestingly, stillbirth rate improved considerably in the Northern region in comparison to the Upper East region, despite the low coverage of facility delivery utilization in the Northern region. The disparity in outcomes of stillbirth between the Upper East and Northern regions appear to suggest that some the underlying context factors may provide explanation to outcomes.

The use of delivery partograph was more of a problem in the Upper East region as one of the respondents puts it, “in the one hospital in the Upper East region, the use of partograph was 0 out of 100”. The analysis of association shows that stillbirth was more likely to be recorded among the ‘free’ maternal health care policy group compared to the no ‘free’ maternal health care policy, which is consistent with the worsening indices as reflected in the rates.

Several factors affect stillbirth and perinatal mortality within the health system which in turn is affected by quality of care dimensions (58). Infection, anemia, preterm delivery and low birth weight are some of the associated factors of stillbirth and these, perhaps are inadequately tackled despite the upsurge of antenatal care uptake and facility level delivery utilization (15,59,60).

Perceived lack of care and attention was espoused by the pregnant women during the focus group discussion. The effect of this is that pregnant women may loss trust in facility level delivery and even turn up late and in complicated state, thus affecting care quality and outcomes (6,61).

The quality of care element is also manifested in the inadequate use of delivery partograph as elicited from the in-depth interviews (62,63). Earlier studies have explored reasons associated with limited or the complete lack of use of delivery partograph citing; inadequate staffing, lack of motivation and the complex nature of delivery partograph (20,64). This current study suggest that

midwives have the tendency of not using the delivery partograph and this may be contributing to the increasing figures of stillbirth.

The findings also show that some pregnant women in the study site hospitals resort to local concoction known as kaligutiem to speed up contraction in labour, unaware of its adverse effect on the unborn child. It is not clear what could possibly be the motivation for pregnant women to want to hastened their labour process, nevertheless, the practice demonstrates some lack of confidence in the health care system's ability to conduct safe delivery of their babies.

While further studies may be required to establish any association between the use of *kaligutiem* and stillbirth outcomes, the account of midwives suggest that pregnant women who take to the local potion are at risk of uterine rapture due to the strong contractions experienced by the users.

Strength and Limitation

This is the first study to compare stillbirth and perinatal mortality outcome following the implementation of the Ghana's 'free' maternal health care policy in 2008. Our definition of stillbirth included born dead of dying within 24 hours after birth. This was necessary to widening the scope and increased the sample size for a much robust estimation.

One other strength of this study was the study use large data sets from Ghana Demographic and Health Survey which is based on multiple cross sectional survey with standardized questionnaire and nationally representative for public health policy evaluation. Another strength of this study was its mix method approach of evaluating not just the mortality outcomes, but also health system challenges which explained the contextual factors affecting the 'free' maternal health care policy

operations and outcomes of late pregnancy. Perhaps the findings of this current study will serve to guide public health policy decisions on stillbirth and perinatal mortality.

In spite of the strengths, the quantitative analysis was based on association using regression models. This is considered a less robust method of determining impact of a policy intervention compared to a quasi-experimental design. Also, the selected regions for the qualitative design were both from northern Ghana, based on the analysis of stillbirth outcomes, thus, limiting the perspective of the pregnant women and service providers relative to the 'free' maternal health care policy.

Conclusion

While the overall perinatal mortality rate declined in 2014, stillbirth rate is still a concern under the current circumstance, thus, suggesting that the 'free' policy implementation is yet to translate meaningfully into reduction in stillbirth outcome. Of a major concern also, is the possibility that expectant mothers are at risk of not completing their required doses of folic acid or iron supplement and probably exposed to sub-standard medicine from the open market due to the intermittent facility level shortages of medicines consumable.

Essentially, access to care policy is not enough in driving the indices of newborn mortality towards desired outcomes, a holistic health system approach which ensures the availability of good quality medicine commodities and well trained human resources, including ultra-sound sonographers are critical to achieving set goals and targets.

Perhaps, the Ministry of Health and Ghana Health Service could adopt responsive approach to maternal health care services in Ghana and this probably, should go beyond bridging financial access gap to ensuring that medicine commodities, health technologies for diagnosis and adequately trained man power are available to support a responsive health system.

Acknowledgment

Demographic and Health Survey Programme funded by the United State Agency for International Development (USAID) is hereby acknowledged for their role in collecting the two rounds of DHS data sets. The authors acknowledge the work of earlier researchers on the study area, from whom literature is drowned to enrich the discussion. We also acknowledge the cooperation and support of Ghana Health Service and the Directors who granted entrée to hospitals and health centers for this study. Finally, we acknowledge the voluntary participants who willingly volunteered to participate in the study.

Author Contributions

Conceptualization: John Azaare

Supervision: Patricia Akweongo, Genevieve Cecilia Aryeteey, Duah Dwomoh

Data collection and cleaning: John Azaare, Stephen Asambo Azari

Methodology: John Azaare, Patricia Akweongo, Genevieve Cecilia Aryeteey, Duah Dwomoh

Formal Analysis: John Azaare, Patricia Akweongo, Genevieve Cecilia Aryeteey, Duah Dwomoh

Writing/Original Draft: John Azaare

Writing/Review & Editing: John Azaare, Patricia Akweongo, Genevieve Cecilia Aryeteey, Duah Dwomoh

Reference

1. Kayode GA, Grobbee DE, Amoakoh-coleman M, Adeleke IT, Ansah E, Groot JAH De, et al. Predicting stillbirth in a low resource setting. BMC Pregnancy Childbirth [Internet]. 2016;1–8. Available from: http://dx.doi.org/10.1186/s12884-016-1061-2

2. Lawn JE, Blencowe H, Waiswa P, Amouzou A, Mathers C, Hogan D, et al. Stillbirths : rates , risk factors , and acceleration towards 2030. Lancet. 2016;387(2):587–603.

3. Dassah ET, Odoi AT, Opoku BK. Stillbirths and very low Apgar scores among vaginal births in a tertiary hospital in Ghana : a retrospective cross-sectional analysis. 2014;1–7.

4. Madhi SA, Briner C, Maswime S, Mose S, Mlandu P, Chawana R, et al. Causes of stillbirths among women from South Africa : a prospective , observational study. Lancet Glob Heal [Internet]. 2019;7(4):e503–12. Available from: http://dx.doi.org/10.1016/S2214-109X(18)30541-2

5. Welaga P, Oduro A, Debpuur C, Aaby P, Ravn H, Andersen A, et al. Fewer out-of-sequence vaccinations and reduction of child mortality in Northern Ghana. Vaccine [Internet]. 2017;35(18):2496–503. Available from: http://dx.doi.org/10.1016/j.vaccine.2017.03.004

6. Roos N. Why do maternal and newborn deaths continue to occur ? Best Pract Res Clin Obstet Gynaecol [Internet]. 2016;36:30–44. Available from: http://dx.doi.org/10.1016/j.bpobgyn.2016.06.002

7. Muglu J, Rather H, Arroyo-manzano D, Id SB, Id JZ, Id ST. Risks of stillbirth and neonatal death with advancing gestation at term : A systematic review and meta-analysis of cohort studies of 15 million pregnancies. 2019;1–16.

8. Moore KA, Simpson JA, Scoullar MJL, Mcgready R, Fowkes FJI. Articles Quantification of the association between malaria in pregnancy and stillbirth : a systematic review and meta-analysis. Lancet Glob Heal [Internet]. 5(11):e1101–12. Available from: http://dx.doi.org/10.1016/S2214-109X(17)30340-6

9. Sugai MK, Gilmour S, Ota E, Shibuya K. Trends in perinatal mortality and its risk factors in Japan: Analysis of vital registration data, 1979–2010. Sci Rep [Internet]. 2017;7(1):1–8. Available from: http://dx.doi.org/10.1038/srep46681

10. Kemp B. Maternal mortality in the UK : an update. Obstet Gynaecol Reprod Med [Internet]. 2016;26(1):26–8. Available from: http://dx.doi.org/10.1016/j.ogrm.2015.11.003

11. Clarke J, Bell C, Coyle D, Stevenson H, Evans G, Mj M, et al. SHARING) SCHEMES IN THE UK A243 UNIVERSITIES TOWARDS THE NEW SAUDI MANDATORY PREFERENCES FOR HEALTH SYSTEM CHARACTERISTICS IN THE A244. Value Heal [Internet]. 2007;12(7):A243–4. Available from: http://dx.doi.org/10.1016/S1098-3015(10)74190-5

12. Bernis L De, Kinney M V, Stones W, Hoope-bender P, Vivio D, Leisher SH, et al. Ending preventable deaths by 2030. Lancet. 2016;387:703–16.

13. Flenady V, Wojcieszek AM, Middleton P, Ellwood D, Erwich JJ, Coory M, et al. Ending

preventable stillbirths 4 Stillbirths : recall to action in high-income countries. 2016;691–702.

14. Ogwulu CB, Jackson LJ, Heazell AEP, Roberts TE. Exploring the intangible economic costs of stillbirth. 2015;1–11.

15. Badimsuguru AB, Nyarko KM, Afari EA, Sackey SO, Kubio C. Determinants of stillbirths in Northern Ghana: a case control study. Pan Afr Med J. 2016;25(Supp 1):18.

16. Tavares F, Silva D, Gonik B, Mcmillan M, Keech C, Dellicour S, et al. Stillbirth : Case definition and guidelines for data collection , analysis , and presentation of maternal immunization safety data ଝ. Vaccine [Internet]. 2016;34(49):6057–68. Available from: http://dx.doi.org/10.1016/j.vaccine.2016.03.044

17. Flenady V, Wojcieszek AM, Ellwood D, Hopkins S, Jaap J, Erwich HM, et al. Classification of causes and associated conditions for stillbirths and neonatal deaths. Semin Fetal Neonatal Med [Internet]. 2017;22(3):176–85. Available from: http://dx.doi.org/10.1016/j.siny.2017.02.009

18. Witter S. An exploratory study of the policy process and early implementation of the free NHIS coverage for pregnant women in Ghana. 2013;(June 2014).

19. Dwomoh D, Agyabeng K, Agbeshie K, Incoom G, Nortey P, Yawson A, et al. Impact evaluation of the free maternal healthcare policy on the risk of neonatal Saharan and infant deaths in four sub- - African countries : a quasi- - experimental design with propensity score Kernel matching and difference in differences analysis. 2020;1–14.

20. Afulani PA. Determinants of stillbirths in Ghana : does quality of antenatal care matter ? BMC Pregnancy Childbirth [Internet]. 2016;1–17. Available from: http://dx.doi.org/10.1186/s12884-016-0925-9

21. Lawn JE, Gravett MG, Nunes TM, Rubens CE, Stanton C, Group R. Global report on preterm birth and stillbirth (1 of 7): defi nitions , description of the burden and opportunities to improve data. BMC Pregnancy Childbirth. 2010;10(Suppl 1):1–22.

22. Arthur E. Wealth and antenatal care use : implications for maternal health care utilisation in Ghana. 2015;1–8.

23. Dassah ET, Adu-sarkodie Y, Mayaud P. Factors associated with failure to screen for syphilis during antenatal care in Ghana : a case control study. 2015;1–9.

24. Manzi A, Munyaneza F, Mujawase F, Banamwana L, Sayinzoga F, Thomson DR, et al. Assessing predictors of delayed antenatal care visits in Rwanda : a secondary analysis of Rwanda demographic and health survey 2010. 2014;1–8.

25. Gibson-helm M, Teede H, Block A, Knight M, East C, Wallace EM, et al. Maternal health and pregnancy outcomes among women of refugee background from African countries : a retrospective , observational study in Australia. 2014;

26. Adu-gyamfi S, Brenya E, Adjei Nuako G. National Health Insurance and Free Maternal Healthcare in Ghana: Responses from Women and Health Workers in Akropong. An Int J Humanit Soc Sci. 2015;2(4):696–737.

27. Azaare J, Akweongo P, Aryeetey GC, Dwomoh D. Impact of free maternal health care

policy on maternal health care utilization and perinatal mortality in Ghana : protocol design for historical cohort study. Reprod Health [Internet]. 2020;1–17. Available from: https://doi.org/10.1186/s12978-020-01011-9

28. Mullan Z. Stillbirths : still neglected ? Lancet Glob Heal. 2016;4:e69.

29. Lavin T, Pattinson R. Does antenatal care timing influence stillbirth risk in the third trimester ? A secondary analysis of perinatal death audit data in South Africa. BJOG. 2018;125:140–7.

30. HERA-Health Partners Ghana. Evaluation of the Free Maternal Health Care Initiative in Ghana. Vol. Vol.1, Health Partners Ghana. Accra & Reet; 2013.

31. Dalinjong PA, Wang AY, Homer CSE. Has the free maternal health policy eliminated out of pocket payments for maternal health services ? Views of women , health providers and insurance managers in Northern Ghana. PLoS One. 2018;1–19.

32. Dzakpasu S, Soremekun S, Manu A, Asbroek G, Tawiah C, Hurt L, et al. Impact of Free Delivery Care on Health Facility Delivery and Insurance Coverage in Ghana ' s Brong Ahafo Region. 2012;7(11).

33. Gabrysch S, Nesbitt RC, Schoeps A, Hurt L, Soremekun S, Edmond K, et al. Does facility birth reduce maternal and perinatal mortality in Brong Ahafo , Ghana ? A secondary analysis using data on 119 244 pregnancies from two cluster-randomised controlled trials. Lancet Glob Heal [Internet]. 2019;7(8):e1074–87. Available from: http://dx.doi.org/10.1016/S2214-109X(19)30165-2

34. Pattinson R, Kerber K, Buchmann E, Friberg IK, Belizan M, Lansky S, et al. Stillbirths 4: how can health systems deliver for mothers and babies ? Lancet [Internet]. 2011;377(9777):1610–23. Available from: http://dx.doi.org/10.1016/S0140-6736(10)62306-9

35. Sakyi EK, Atinga RA, Adzei FA. Managerial problems of hospitals under Ghana's National Health Insurance Scheme. Clin Gov An Int J. 2012;17(3):178–90.

36. Ahenkan A, Azaare J. Managerial implications of Hospitals with Delayed Reimbursement of National Health Insurance Claims: The case of Two Hospitals in Ghana. African J Manag Res. 2018;25:62–75.

37. Ahenkan A, Azaare J. Managerial implications of delayed reimbursement of National Health Insurance Claims: The case of two hospital in Northern Ghana. African J Manag Res. 2018;25:62–75.

38. Engmann C, Walega P, Aborigo RA, Adongo P, Moyer CA, Lavasani L, et al. Stillbirths and early neonatal mortality in rural Northern Ghana. Trop Med Int Heal. 2012;17(3):272–82.

39. Haruyama R, Gilmour S, Ota E, Abe SK, Rahman MM, Nomura S, et al. Causes and risk factors for singleton stillbirth in Japan: Analysis of a nationwide perinatal database, 2013-2014. Sci Rep [Internet]. 2018;8(1):1–10. Available from: http://dx.doi.org/10.1038/s41598-018-22546-9

40. Aminu M, Bar-zeev S, Broek NVANDEN. Cause of and factors associated with stillbirth : a systematic review of classification systems. AOGS. 2017;96:519–28.

41. Akazili J, Welaga P, Bawah A, Achana FS, Oduro A, Awoonor-Williams JK, et al. Is Ghana's pro-poor health insurance scheme really for the poor? Evidence from Northern Ghana. BMC Health Serv Res. 2014;14.

42. GSS. Population & housing census report: Children , adolescents & young people in Ghana. Ghana Statistical Service. Accra; 2010.

43. NHIA. National Health Insurance Authority 2013 Annual Report. Accra; 2013.

44. Akazili J, Welaga P, Bawah A, Achana FS, Oduro A, Awoonor-williams JK, et al. Is Ghana ' s pro-poor health insurance scheme really for the poor ? Evidence from Northern Ghana. 2015;1–9.

45. Creswell JW. Qualitative Inquiry & Research Design: Choosing among Five Approaches. Thousand Oaks, CA: SAGE. 3rd ed. SAGE; 2013.

46. Bhutta ZA, Das JK, Rizvi A, Gaff MF, Walker N, Horton S, et al. Maternal and Child Nutrition 2 Evidence-based interventions for improvement of maternal and child nutrition : what can be done and at what cost ? 2013;382.

47. Mosley H, Chen LC. Frameworks for studying the determinants of child survival. 2003;81(02):138–9.

48. Ibrahim A, Maya ET, Donkor E, Agyepong IA, Adanu RM. Perinatal mortality among infants born during health user-fees (Cash & Carry) and the national health insurance scheme (NHIS) eras in Ghana : a cross-sectional study. BMC Pregnancy Childbirth [Internet]. 2016;1–8. Available from: http://dx.doi.org/10.1186/s12884-016-1179-2

49. Blanchet NJ, Fink G. The effect of Ghana's National Health Insurance Scheme on Health Care Utilization. 2012;46(2).

50. Nuamah GB, Agyei-baffour P, Mensah KA, Boateng D, Quansah DY, Dobin D, et al. Access and utilization of maternal healthcare in a rural district in the forest belt of Ghana. 2019;5:1–11.

51. International S, Health R, June N. Removal of User Fees Associated with Increases In Delivery at Facilities. 2018;41(2):109–10.

52. Gibbons K, Gordon A, Smith GC, Ezzati M, Flenady V, Fretts R, et al. Major risk factors for stillbirth in high-income countries: a systematic review and meta-analysis. Lancet [Internet]. 2011;377(9774):1331–40. Available from: http://dx.doi.org/10.1016/S0140-6736(10)62233-7

53. Aminu M, Bar-zeev S, White S, Mathai M, Broek N Van Den. Understanding cause of stillbirth : a prospective observational multi-country study from sub-Saharan Africa. BMC Pregnancy Childbirth. 2019;7:1–10.

54. Mensah KB, Opoku-agyeman K, Ansah C. Antibiotic use during pregnancy : a retrospective study of prescription patterns and birth outcomes at an antenatal clinic in rural Ghana. 2017;4–10.

55. Qu Y, Chen S, Pan H, Zhu H, Yan C, Zhang S, et al. Risk factors of stillbirth in rural China: A national cohort study. Sci Rep [Internet]. 2019;9(1):1–8. Available from: http://dx.doi.org/10.1038/s41598-018-35931-1

56. Atinga R a. Healthcare quality under the National Health Insurance Scheme in Ghana: Perspectives from premium holders. Int J Qual Reliab Manag [Internet]. 2012;29(2):144–61. Available from: http://www.scopus.com/inward/record.url?eid=2-s2.0-84856384812&partnerID=40&md5=6fddbc16acb6d1b2c9c6ebf337055e3f

57. Atinga RA, Abekah-nkrumah G, Domfeh KA. patient satisfaction Managing healthcare quality in Ghana : a necessity of patient satisfaction. 2011;

58. Atinga RA, Abekah-Nkrumah G, Domfeh KA. Managing healthcare quality in Ghana : a necessity of patient satisfaction. Int J Health Care Qual Assur. 2011;24(7):548–63.

59. Goodman D, Ramaswamy R, Jeuland M, Srofenyoh E, Engmann C, Olufolabi A, et al. Evaluating the Cost-Effectiveness of an Integrated Program to Reduce Maternal and Neonatal Mortality in Ghana. Ann Glob Heal [Internet]. 2017;83(1):199–200. Available from: http://dx.doi.org/10.1016/j.aogh.2017.03.495

60. Neogi SB, Sharma J, Negandhi P, Chauhan M, Reddy S. Risk factors for stillbirths : how much can a responsive health system prevent ? 2018;1–10.

61. Allanson ER, Pattinson RC. Lessons from the field: Quality-of-care audits and perinatal mortality in South Africa. Bull World Heal Organ. 2015;(March):424–8.

62. Asibong U, Okokon BI, Oku A, Opiah M, Essien EJ, Monjok E. The use of the partograph in labor monitoring : a cross-sectional study among obstetric caregivers in General Hospital ,. Int J Womens Health. 2014;6:873–80.

63. Yisma E, Dessalegn B, Astatkie A, Fesseha N. Completion of the modified World Health

Organization (WHO) partograph during labour in public health institutions of Addis Ababa , Ethiopia. Reprod Health. 2013;10(23):1–7.

64. Zere E, Kirigia JM, Duale S, Akazili J. Inequities in maternal and child health outcomes and interventions in Ghana. BMC Public Health [Internet]. 2012;12(1):252. Available from: http://www.biomedcentral.com/1471-2458/12/252

Figure Click here to access/download;Figure;Fig 1.docx

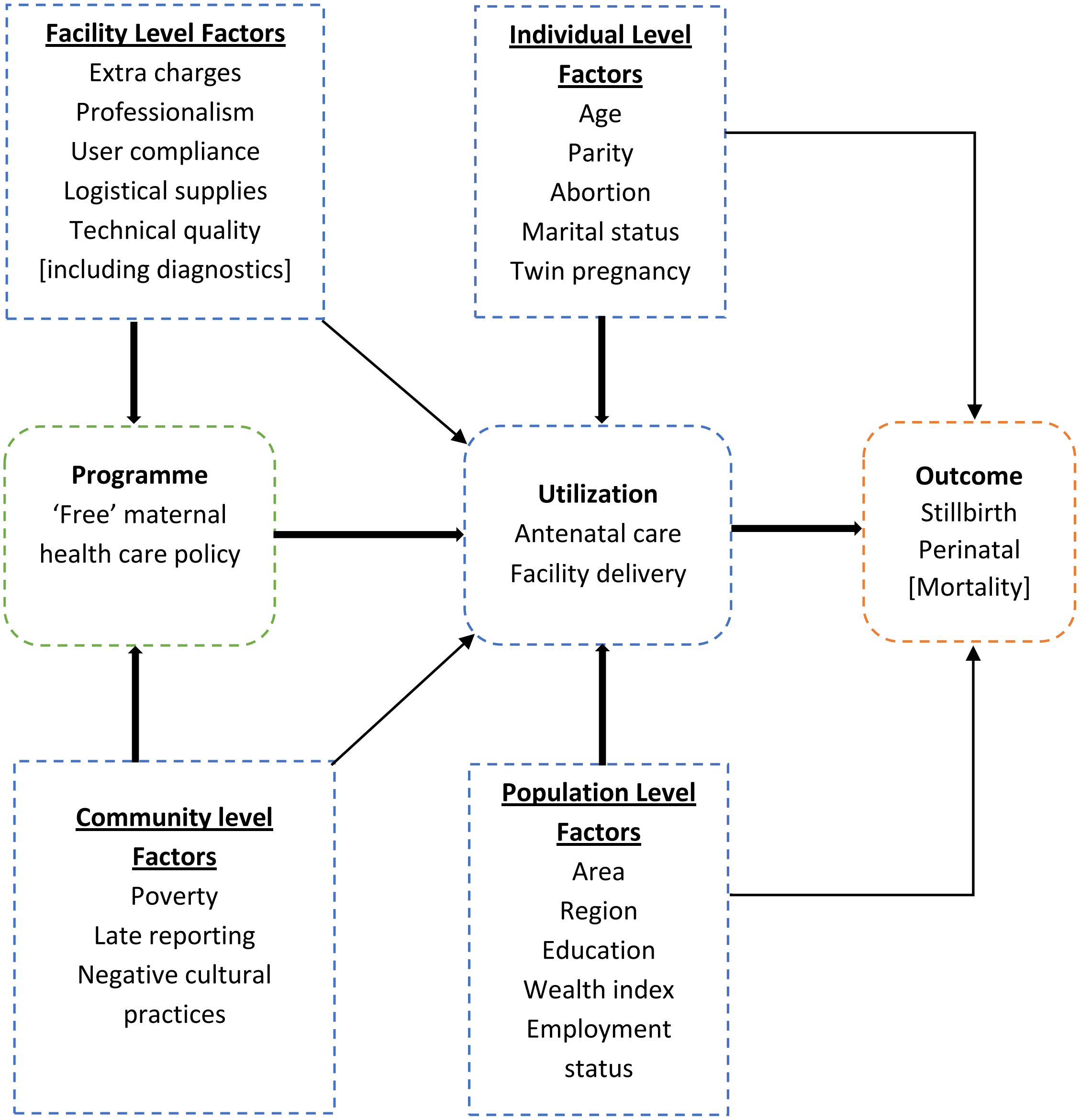

Figure 1: Moderating factors of stillbirth and perinatal mortality in a multifaceted relationship

Figure

Click here to access/download;Figure;Fig 2.docx

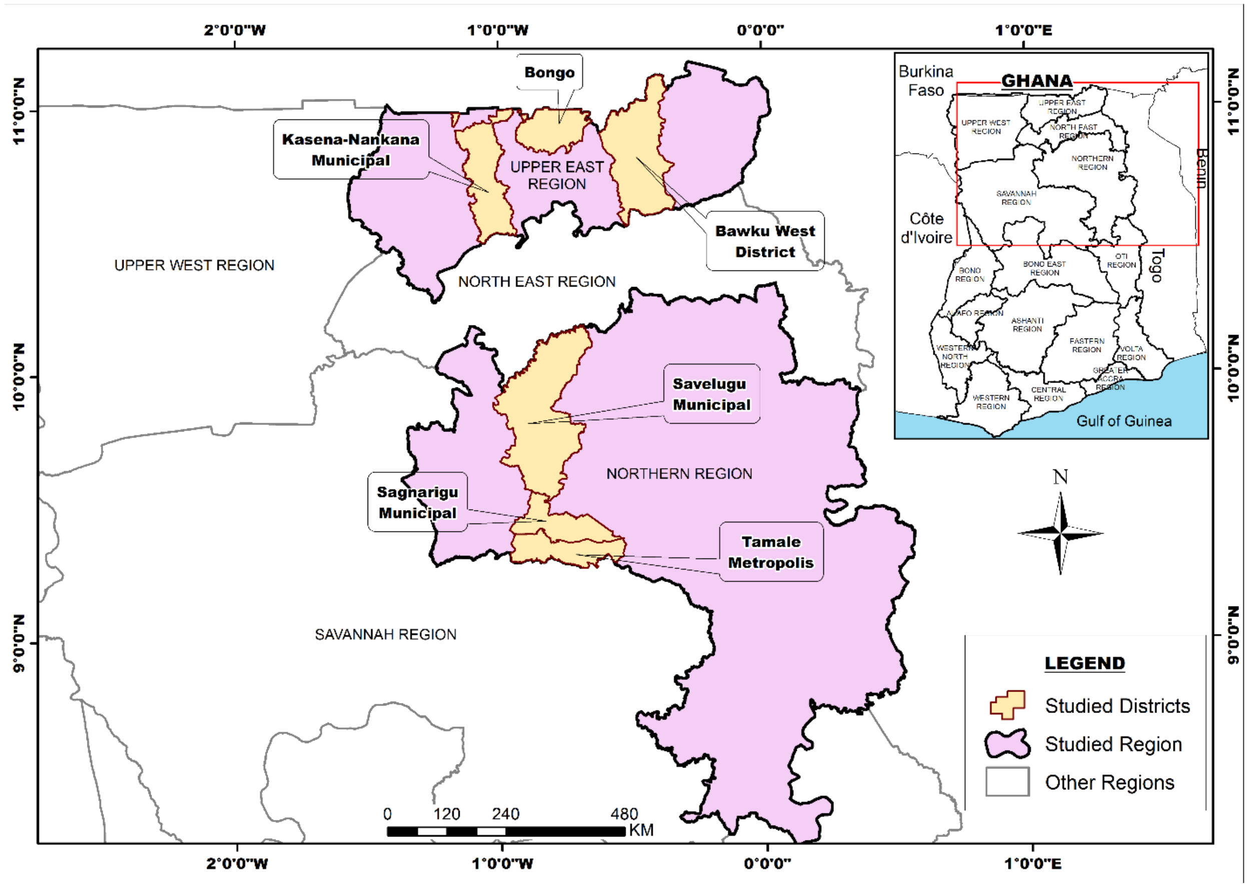

Figure 2. Map of Ghana showing the Upper East and Northern regions and the selected districts

Click here to access/download
Supporting Information
IDI and FGD guide.pdf

Click here to access/download
Supporting Information
RDH.pdf
