# Supplementary material for: Evaluating the impact of maternal health care policy on stillbirth and perinatal mortality in Ghana; a mixed method approach using two rounds of Ghana demographic and health survey data sets and qualitative design technique
Source: PLoS One. 2022 Sep 29;17(9):e0274573. doi: 10.1371/journal.pone.0274573 (PMC9521900; doi:10.1371/journal.pone.0274573)
Supplement: Attachment — Submitted filename: Response to reviewers.docx [file pone.0274573.s002.docx]

The Academic Editor

PLOS ONE Journal

Dear Editor,

**RE: RESPONSE TO REVIEWERS: PONE-D-21-35524 - EVALUATING THE IMPACT OF MATERNAL HEALTH CARE POLICY ON STILLBIRTH AND PERINATAL MORTALITY IN GHANA: A MIX METHOD APPROACH USING TWO ROUNDS OF GHANA DEMOGRAPHIC AND HEALTH SURVEY DATA SETS AND QUALITATIVE DESIGN TECHNIQUE**

1. The manuscript formatting has been revised per PLOS journal requirements including Tables and Figures. We are willing to revise further if necessary, any aspect that does not meet the requirement of the journal. However, this may have to be pointed out clearly to us to guide us.
2. The study objective has been restated to make it much clearer than before.
3. The size of the manuscript has also been reduced particularly in the introduction and discussion sections:
   a. the contextual definition sub-section has been taken out and key definition incorporated into the introduction.
   b. the formula for the calculation of stillbirth and perinatal mortality ratios has also been removed from the manuscript.
   c. the Tables has been reduced and others merged as necessary. We now have 5 Tables instead of 7 as contained in the original submission.
4. You noted that some aspects of this manuscript appear to have been published somewhere else. This is not exactly clear to us. However;
   a. the original protocol design of the study was published by BMC reproductive health journal and assigned DOI (<https://doi.org/10.1186/s12978-020-01011-9>) which was just the methodology of the full protocol at its design stage and did not include any results. The conceptual framework in the protocol appears similar to the one in the current manuscript, but not the same. There has been a significant modification as the study progressed which is what has been submitted in the current manuscript.
   b. In preparing this manuscript, aspects of the qualitative results were presented by research gate as a pre-publication text (not peer-reviewed), usually meant to gather useful comments to enrich the manuscript (and does not constitute a publication of our current results) and thus, the wording may be similar. Accordingly, we can confirm that no part of this manuscript results has been published in any journal as a research finding and should be considered for publication in PLOS Journal as an original piece of work.
5. *(****We also acknowledge the cooperation and support of Ghana Health Service and the Directors who granted entrée to hospitals and health centres for this study****.)*
6. The above statement (item 5) was quoted to us, and intimating that we may have received funding support from Ghana Health Service or another agency. Perhaps, we did not convey the appropriate impression in the choice of words and terms such as “support of Ghana Health Service…”. However, for the avoidance of doubt, no funding support was received from Ghana Health Service or any agency of local or international in nature regarding this study. This manuscript is part of the PhD research work of the first author during his candidature in the University of Ghana School of Public Health, where the 2^nd^, 3^rd^ and 4^th^ authors are faculty and supervising committee members of the 1^st^ author. The first author did receive tuition fee support (not research) from the Ghana Education Trust Fund (GetFUND) and this has been duly acknowledged in the manuscript. We are willing to modify our statement if the journal finds the GetFUND tuition fee as funding support for the research work.
7. Figure 2 (Fig. 2) attached in the manuscript is an original map professionally constructed for this study and about the selected study site from two regions of Ghana. The map is an original work designed for it purpose following the commencement of the study and has neither been used by anyone nor published anywhere before. To this end, we do not find the need to follow your guideline on copyright concerning third party use of a figure (the map), as this does not apply to us in this case. We are willing to remove the particular figure (Fig. 2) if there is evidence to the contrary.
8. The aspect of the ethical statement has been revised accordingly in the method section of the manuscript. The full name of the ERB, Ghana Health Service Ethical Review Board has been stated under the sub-section **‘Ethical consideration’.**
9. All Tables and Figures have been duly referred to i.e. Table 7 which was cited as not referred to has been corrected. The particular Table was referenced as multiple Tables in the manuscript. However, in the revised version, Table 7 (now Table 6) is attached as a supplementary file.
10. The figures (Fig 1 and 2) have been uploaded to **the Preflight Analysis and Conversion Engine (PACE) digital diagnostic** accordingly to meet PLOS requirements.
11. Humbly submitted.
