# Supplementary material for: Evaluating the impact of maternal health care policy on stillbirth and perinatal mortality in Ghana; a mixed method approach using two rounds of Ghana demographic and health survey data sets and qualitative design technique
Source: PLoS One. 2022 Sep 29;17(9):e0274573. doi: 10.1371/journal.pone.0274573 (PMC9521900; doi:10.1371/journal.pone.0274573)
Supplement: Attachment — Submitted filename: Reviewers comments_14052022.docx [file pone.0274573.s004.docx]

**Review comments**

**Manuscript Title: Evaluating the impact of maternal health care policy on stillbirth and perinatal mortality in Ghana: a mix method approach using two rounds of Ghana demographic and health survey data sets and qualitative design technique**

**Abstract**

1. **Background:** The current percentage decline from 2000-2019 is 35% with the annual rate of reduction being 2% according to WHO/UNICEF 2020. This is still impressive but maybe perhaps its not enough to achieve the 2030 targets of 12/1000 stillbirth per live births or less. The authors need to provide these facts.
2. **Methods:** How mixed were they? Were the qualitative interviews interrogating the observed patterns from the quantitative results or it was simply parallel where we do not expect results to feed into each other. Clarification about this is needed.
3. **Results:** how are the results integrated? For example were the observed differences between the 2008 and 2014 results solely explained by absence of folic acids and multivitamins as well as unauthorised use of oxytocin or these work through pathways to result into an eventual stillbirth?. The authors need to bring this out
4. **Conclusion:**  women use of traditional herbs is not part of the policy that was being evaluated. Perhaps it’s a reflection of poor sensitization by the policy and it should be reflected as such. Reporting use of traditional herbs when it was not part of the study objective is not called for.
5. **Key works;**  “policy evaluation” is missing and yet crucial

**Introduction**

1. Stillbirth and perinatal mortality are part of the maternal and child health indicators which are very sensitive health system indicators reflecting negatively on the overall performance of the health systems. This needs to come out clearly.
2. Stillbirth and perinatal mortality have received relatively good policy attention. Eg newborn health has received renewed attention and it has the potential of addressing perinatal mortality. Stillbirth too has received relative attention. The ENAP set targets for national governments to adopt towards addressing the two. You should reflect on what Ghana has done in line to achieve the ENAP targets.
3. The global burden of 2.6 stillbirth is an old statistic. The current burden is 2 million annual stillbirth according to UNICEF/WHO report titled “The neglected tragedy; the global burden of stillbirth 2020. The authors need to use Up to date references.
4. Technically, the WHO defines stillbirth as intrauterine death after conception”. This statement is not true. The definition only applies to foetal death after 28 weeks. Revise as appropriate.
5. “Access to maternal health care is proven to reduce stillbirth”. It is not access to any maternal healthcare but rather “ quality and timely maternal healthcare” you need to revise this to reflect the recommendation
6. Related to the above, the authors need to highlight the global burden, regional picture and country status to bring out the problem clearly in a funnel-like approach.
7. Did that policy explicitly say that it targeted to address stillbirth and perinatal mortality? You need to reflect it in the introduction.
8. Policy components of the free maternal healthcare policy need to be highlighted for the reader. Right now it is not known.
9. Other than the social health insurance, the main maternal and child health intervention since 2000 has been the MDGs with specific goals for maternal and child health whose intervention impacts directly on the indicators you are studying in this study. This relationship needs to come out explicitly.
10. As early stated, other than quoting the 2015 statistics, its better to use recent data from the 2020 UNICEF/WHO report.
11. Also, instead of quoting Nigerian data it would be important to quote the stillbirth burden in Ghana straight away.
12. The definition of stillbirth is not clear; key issues to note are the 28 weeks threshold as the official position of WHO, and birth with no sign of life either fresh or macerated. This needs to come out explicitly and when using the official definition of WHO, its better to use their own sources.
13. Other definitions which stretch to less than 28 weeks of gestation are not official but rather country and study specific definitions based on period of viability.
14. There are contextual factors which need to come out clearly in the background. For example, the MDGs were a cornerstone for improvement of maternal and child health services globally. Also, the UN secretary general strategy Every Woman Every Child which was operationalised through the Every Newborn Action Plan (ENAP) need to be highlighted when investigating stillbirth. They are the back born for the renewed interest in global stillbirth campaigns.
15. An explanation of the “free maternal health policy within the national health insurance scheme needs to be done in the introduction. Was it only aimed at improving access through addressing the healthcare costs? Or there are maternal health interventions within such as increased access to EmOC, MPDSR among others.
16. “Studies have shown that maternal age, rural/urban area of residence”. This paragraph ignores the important contribution of the health systems factors in addressing stillbirth risks and yet the said policy was implemented within the health systems. It would be important to strike a balance by highlighting the health systems related factors too.
17. *Contextual definitions:* Stillbirth; kindly check this definition. It lacks the basics of what would constitute a stillbirth such as after the 28 weeks cut off.
18. *Free maternal healthcare policy:* This is simplistic. I suggest an elaboration of the ingredients of this policy and the aspects of maternal health that were covered. Its coverage within the Ghanian population and any other important information.
19. Page 13 “ local contest” did you want to mean “context”? if so change as appropriate.
20. “we analyse the outcomes of stillbirth and perinatal deaths among mothers in2008, when the policy had just started, compared to 2014 when the policy was fully rolled out”. This should speak directly to the title of the paper and objective of the study in the introduction of your abstract.

**Methods**

1. Study design; the section is rather speaking more to the study setting other than the design. Be explicit and intentioned right away.
2. Study setting; some information given is irrelevant to this paper, rather give information that adds value. For example the geographical location may not be that needed, instead data on stillbirth and perinatal mortality may be more important here.
3. The sampling procedure for the qualitative component is missing.
4. Details about the qualitative tool are missing. It would be important to take the reader through the kind of information the tool elicited.
5. Instead of attaching the ethics review letter, better quote the reference number for the protocol from the ethics committee.
6. No mention of the inclusion and exclusion criteria for FGD participants. It needs to come out.
7. On the qualitative data analysis, how was the codebook developed, who coded the data? How many people were involved in the analysis?
8. How was saturation attained
9. Attribution of the reduction to the policy
10. The authors need to clarify explicitly how the two methods of data collection were integrated and informed each other. They should also state if this did not happen and why.
11. I would suggest that you follow the COREQ Checklist to report on your qualitative findings better.

**Results**

1. “On risk of stillbirth, pregnancies in the ‘Free’ Maternal Health Policy (FMHCP) group were 1.64 times more likely to result in stillbirth compared to the no FMHCP group”. This needs to come out explicitly in the methods section how these groups were created.
2. Qualitative study participants: the unit of analysis for the FGDs is the group. Therefore, when describing your study participants, its better to refer to how many FGDs were conducted other than the total number of participants within those FGDs.
3. “The majority of pregnant women respondents had given birth previously, 41 (90.1%). Only 4 (8.9%) pregnant women participants were having children for the first time”. To me these appear to belong to the same group unless if you want to say that some were pregnant at the time of the interview and reference is being made to the index pregnancy. Revise statement to bring out clarity.
4. Table 6: the column on average duration is less significant. This description should be in the narrative and refer to the average duration of each interview. In its current form it appears to be the total duration of all interviews in each category. Kindly revise as appropriate.
5. “…A few cases also dodge the hospital may they have two previous CS and knows that if they come to the hospital, there will be CS, so they avoid the hospital, when there are complications, then they quickly come...” (Doctor 1, IDI, UER). Start to use “signposts” in your write up. Whereas the introductory statement was speaking to increased number of macerated stillbirth, this statement seem to be off. Revise as appropriate.
6. “…and when they come we manage them at postnatal care…. they say they didn’t know that labour had started, some say they don’t want the examination. One woman was frank, she said when they come, they put fingers on her vagina and that one she doesn’t like it…” (Doctor 2, IDI, UER). Refer to comment above for this quotation.
7. Service providers also observed a culture of little or no attention to stillbirth issues. Public or media lack of attention on stillbirth was cited as a principal reason compared to maternal mortality. On the other hand, pregnant women midwives’ snobbish attitude to them during labour, as a contributory factor to the rising stillbirths. The following quotes explains further;. Was this part of your study objective or scope of the study? To me it appears not.
8. “Use of local herbs for ‘rapid’ uterine contraction” this can’t be a subtheme of the study since its not part of the policy. It is rather inadequate implementation of the policy that is resulting into this. The write up should reflect that. Its either poor access to available services or poor enforcement of the policy that is resulting into this
9. The qualitative findings only talk about the high numbers of stillbirth which have been explained to be caused by poor use of partograph for pregnancy monitoring and shortage of medicines. I highly doubt this is comprehensive and can solely explain the quantitative findings. More exploration of the qualitative data for more reasons to explain the high numbers.
10. “Service providers are of the view that the increase in utilization puts pressure”. Ensure to write the results section in past tense since activities and comments happened sometime back.
11. On the other hand, pregnant women midwives’ snobbish attitude to them during labour”. Revise sentence as appropriate.
12. Just so to confirm are these FGD quotes from mothers that experienced a stillbirth? If so it should be reported as such and if not you need to clarify in the methodology section.
13. Due to the facility level shortages, pregnant women regularly asked to purchased medicines outside of the health facility”. Revise statement as appropriate, it is not clear in its current form.
14. “This was not only a problem to the pregnant women, but also frustrates the quality of processes of the health care professionals” use past tense as appropriate.

**Discussion**

1. Revise the discussion section accordingly after effecting changes in the results section
2. “improvement in perinatal mortality” is it improvement in perinatal mortality or reduction in perinatal mortality?.

**Conclusion**

1. Revise the discussion section accordingly after effecting changes in the results section
