# Supplementary material for: Evaluating the impact of maternal health care policy on stillbirth and perinatal mortality in Ghana; a mixed method approach using two rounds of Ghana demographic and health survey data sets and qualitative design technique
Source: PLoS One. 2022 Sep 29;17(9):e0274573. doi: 10.1371/journal.pone.0274573 (PMC9521900; doi:10.1371/journal.pone.0274573)
Supplement: Attachment — Submitted filename: Response to Reviewers.docx [file pone.0274573.s005.docx]

Dear EDITOR,

RESPONSE TO REVIEWERS COMMENTS

**Manuscript Title: Evaluating the impact of maternal health care policy on stillbirth and perinatal mortality in Ghana: a mix method approach using two rounds of Ghana demographic and health survey data sets and qualitative design technique**

**Abstract**

All comments under the ABSTRACT sub-section have been addressed as follows;

1. **Background:** This has been revised appropriately and the WHO/UNICEF, 2020 report referenced.
2. **Methods:** The quantitative data was analysed first, then the findings necessitated the qualitative study. The IDIs, especially the expert views were born out of the findings of the quantitative findings. The interview guide is attached as supplementary file.
3. **Results:** Although the results of the qualitative findings provide a certain perspective to the quantitative finings, this study does not seek to establish causal inferences between the results of the quantitative analysis and the qualitative interviews. Its aim was to seek understanding within what contest the ‘free’ policy was expected to produce the desire impact.
4. **Conclusion:**  The qualitative results did not report exactly what was or what was not part of the ‘free’ maternal health care policy. It reported on what context factors affected the ‘free’ policy implementation and therefore, a consequence the stillbirth outcomes, despite the ‘free’ policy. This has been clarified in the introduction and mothed section.
5. **Key works;** “policy evaluation” been included accordingly.

**Introduction**

1. **Comment:** Stillbirth and perinatal mortality are part of the maternal and child health indicators which are very sensitive health system indicators reflecting negatively on the overall performance of the health systems. This needs to come out clearly.

**Response:** Sentence has accordingly been revised.

2. **Comment:** Stillbirth and perinatal mortality have received relatively good policy attention. E.g. new born health has received renewed attention and it has the potential of addressing perinatal mortality. Stillbirth too has received relative attention. The ENAP set targets for national governments to adopt towards addressing the two. You should reflect on what Ghana has done in line to achieve the ENAP targets.

**Response:** Although not explicitly stated, the ‘free’ maternal health care is implicitly implemented to achieve ascertain level of access to antenatal care and facility delivery utilization, which essentially feed into the purpose of ENAP. This has been explained in the opening introduction leading to stating the purpose of the study.

3. **Comment:** The global burden of 2.6 stillbirth is an old statistic. The current burden is 2 million annual stillbirths according to UNICEF/WHO report titled “The neglected tragedy; the global burden of stillbirth 2020. The authors need to use Up to date references.

**Response**: This has been revised.

4. **Comment:** Technically, the WHO defines stillbirth as intrauterine death after conception”. This statement is not true. The definition only applies to foetal death after 28 weeks. Revise as appropriate.

**Response:** Comments revised as suggested.

5. **Comment:** “Access to maternal health care is proven to reduce stillbirth”. It is not access to any maternal healthcare but rather “quality and timely maternal healthcare” you need to revise this to reflect the recommendation.

**Response:** “Quality and timely access” added to said sentence as recommended.

6. **Comment:** Related to the above, the authors need to highlight the global burden, regional picture and country status to bring out the problem clearly in a funnel-like approach.

**Response**: revised.

7. **Comment:** Did that policy explicitly say that it targeted to address stillbirth and perinatal mortality? You need to reflect it in the introduction.

**Response:** The policy included comprehensive antenatal care, facility level delivery and post-delivery care up to 10 days. During this period care, one of the gains expected by implication os reduced stillbirths and perinatal death. This is the bases for this study, to evaluate the ‘free’ policy contribution to late pregnancy outcomes and new-born care.

8. **Comment:** Policy components of the free maternal healthcare policy need to be highlighted for the reader. Right now it is not known.

**Response:** This has been revised in the introductory section.

9. **Comment:** Other than the social health insurance, the main maternal and child health intervention since 2000 has been the MDGs with specific goals for maternal and child health whose intervention impacts directly on the indicators you are studying in this study. This relationship needs to come out explicitly.

**Response:** Since MDGs were reviewed to SDGs, this is perhaps, a more appropriate reference to make which is what we made in our introduction section.

10. **Comment:** As early stated, other than quoting the 2015 statistics, it’s better to use recent data from the 2020 UNICEF/WHO report.

**Response**: The UNICEF/WHO reported has been used to update the figures and appropriated referenced.

11. **Comment:** Also, instead of quoting Nigerian data it would be important to quote the stillbirth burden in Ghana straight away.

**Response:** Ghana data used instead of Nigeria.

12. **Comment:** The definition of stillbirth is not clear; key issues to note are the 28 weeks’ threshold as the official position of WHO, and birth with no sign of life either fresh or macerated. This needs to come out explicitly and when using the official definition of WHO, it’s better to use their own sources.

**Response:** Statement revised.

13. **Comment:** Other definitions which stretch to less than 28 weeks of gestation are not official but rather country and study specific definitions based on period of viability.

**Response:** Definitions has been edited and revised.

14. **Comment:** There are contextual factors which need to come out clearly in the background. For example, the MDGs were a cornerstone for improvement of maternal and child health services globally. Also, the UN secretary general strategy Every Woman Every Child which was operationalised through every New Born Action Plan (ENAP) need to be highlighted when investigating stillbirth. They are the back born for the renewed interest in global stillbirth campaigns.

**Response:** Comments appropriately incorporated.

15. **Comment**: An explanation of the “free maternal health policy within the national health insurance scheme needs to be done in the introduction. Was it only aimed at improving access through addressing the healthcare costs? Or there are maternal health interventions within such as increased access to EmOC, MPDSR among others.

**Response:** The ‘free’ maternal health care policy explained and the national health insurance explained.

16. **Comment:** “Studies have shown that maternal age, rural/urban area of residence”. This paragraph ignores the important contribution of the health systems factors in addressing stillbirth risks and yet the said policy was implemented within the health systems. It would be important to strike a balance by highlighting the health systems related factors too.

**Response:** indeed, the policy is being implemented with certain health systems factors, which was the focus of the qualitative study. Accordingly, the sentence has been revised.

17. **Comment:** *Contextual definitions:* Stillbirth; kindly check this definition. It lacks the basics of what would constitute a stillbirth such as after the 28 weeks cut off.

**Response:** Definition revised.

18. **Comment**: Free maternal healthcare policy: This is simplistic. I suggest an elaboration of the ingredients of this policy and the aspects of maternal health that were covered. Its coverage within the Ghanaian population and any other important information.

**Response:** This has been revised and scope and package included in the introduction section.

19. **Comment**: Page 13 “local contest” did you want to mean “context”? if so change as appropriate.

**Response:** Yes, this refers to ‘context. Appropriately edited.

20. **Comments:** “we analyse the outcomes of stillbirth and perinatal deaths among mothers in2008, when the policy had just started, compared to 2014 when the policy was fully rolled out”. This should speak directly to the title of the paper and objective of the study in the introduction of your abstract.

**Response:** Comments revised accordingly.

**Methods**

1. **Comment:** Study design; the section is rather speaking more to the study setting other than the design. Be explicit and intentioned right away.

**Response:** This comment has been addressed.

2. **Comment:** Study setting; some information given is irrelevant to this paper, rather give information that adds value. For example, the geographical location may not be that needed, instead data on stillbirth and perinatal mortality may be more important here.

**Response:** Data on stillbirth for the qualitative study sites have been included.

3. **Comment:** The sampling procedure for the qualitative component is missing.

**Response:** Perhaps, this was clear enough. It has been revised accordingly.

4. **Comment:** Details about the qualitative tool are missing. It would be important to take the reader through the kind of information the tool elicited.

**Response**: A little more detail has been added on the qualitative information. Tools also attached as supplementary file.

5. **Comment:** Instead of attaching the ethics review letter, better quote the reference number for the protocol from the ethics committee.

**Response:** protocol number quoted under the section ‘ethical consideration’

6. **Comment:** No mention of the inclusion and exclusion criteria for FGD participants. It needs to come out.

**Response:** This had been revised to include inclusion and exclusion criteria of FDGs.

7. **Comments:** On the qualitative data analysis, how was the codebook developed, who coded the data? How many people were involved in the analysis?

**Response:** The first author analysed the qualitative data and supervised by the second and third authors. Coding essentially was guided by the deductive themes of stillbirth, and the context factors affecting maternal health care utilization under the ‘free’ policy and how they affect ‘stillbirth’ outcome. The emerged significant statements were grouped as inductive sub-themes and reported as the study results with verbatim quotes. Statements were reviewed thoroughly for significance through multiple reading and grouped as affecting quality of care and perhaps negatively influencing stillbirth.

8. **Comment:** How was saturation attained?

**Response:** When subsequent IDIs added no particular new information, we determined that saturation was reached, and suspended interviews. However, FGDs continued as planned and all 8 groups of FGDs were carried out and recorded views transcribed analysed in unison.

9. **Comment:** Attribution of the reduction to the policy

**Response:** This comment is not particularly clear. However, the study found that perinatal mortality rate declined between 2008 and 2014, while stillbirth rate increased within the same period. Yet, both perinatal mortality and stillbirth were more likely to occur in the ‘free’ maternal health care policy group compared to the no ‘free’ maternal health care policy group.

10. **Comments:** The authors need to clarify explicitly how the two methods of data collection were integrated and informed each other. They should also state if this did not happen and why.

**Response**: data collection and integration has been revised under the methods section

11. **Comment**: I would suggest that you follow the COREQ Checklist to report on your qualitative findings better.

**Response:** Although, the Consolidated Criteria for Reporting Qualitative research (COREQ) was not particularly used in the study, nonetheless, aspects in the manuscript have addressed issues the requirements of the COREQ checklist. The qualitative study conducted mainly by the first author, as a PhD candidate of the University of Ghana School of Public Health, under a thesis supervisory committee led by the second author, received ethical approval from the Ghana Health Service Ethical Review Committee (number quoted in main test of manuscript). Analysis was content in nature, where sub-themes were constructed to report the study results.

**Results**

1. **Comment:** “On risk of stillbirth, pregnancies in the ‘Free’ Maternal Health Policy (FMHCP) group were 1.64 times more likely to result in stillbirth compared to the no FMHCP group”. This needs to come out explicitly in the methods section how these groups were created.

**Response:** Group creation has been revised accordingly in the design section. Also, Table 2 has been revised to report the values of the two groups.

2. **Comment:** Qualitative study participants: the unit of analysis for the FGDs is the group. Therefore, when describing your study participants, its better to refer to how many FGDs were conducted other than the total number of participants within those FGDs.

**Response:** descriptive section of FDGs revised appropriately.

3. **Comment:** “The majority of pregnant women respondents had given birth previously, 41 (90.1%). Only 4 (8.9%) pregnant women participants were having children for the first time”. To me these appear to belong to the same group unless if you want to say that some were pregnant at the time of the interview and reference is being made to the index pregnancy. Revise statement to bring out clarity.

**Response:** Yes, we agree with your comment. The section/sentence has be revised.

4. **Comment:** Table 6: the column on average duration is less significant. This description should be in the narrative and refer to the average duration of each interview. In its current form it appears to be the total duration of all interviews in each category. Kindly revise as appropriate.

**Response:** We agree with the reviewer. The particular column has be deleted appropriately.

5. **Comment:** “…A few cases also dodge the hospital may they have two previous CS and knows that if they come to the hospital, there will be CS, so they avoid the hospital, when there are complications, then they quickly come...” (Doctor 1, IDI, UER). Start to use “signposts” in your write up. Whereas the introductory statement was speaking to increased number of macerated stillbirth, this statement seem to be off. Revise as appropriate.

**Response:** Statement/section revised appropriately.

6. **Comment**: “…and when they come we manage them at postnatal care…. they say they didn’t know that labour had started, some say they don’t want the examination. One woman was frank, she said when they come, they put fingers on her vagina and that one she doesn’t like it…” (Doctor 2, IDI, UER). Refer to comment above for this quotation.

**Response:** Also revised.

7. **Comment:** Service providers also observed a culture of little or no attention to stillbirth issues. Public or media lack of attention on stillbirth was cited as a principal reason compared to maternal mortality. On the other hand, pregnant women midwives’ snobbish attitude to them during labour, as a contributory factor to the rising stillbirths. The following quotes explains further. Was this part of your study objective or scope of the study? To me it appears not.

**Response:** Yes, these emerged from the IDIs, as some of the contextual factors that bothers on stillbirth relative the ‘free’ policy in practice.

8. **Comment:** “Use of local herbs for ‘rapid’ uterine contraction” this can’t be a subtheme of the study since its not part of the policy. It is rather inadequate implementation of the policy that is resulting into this. The write up should reflect that. Its either poor access to available services or poor enforcement of the policy that is resulting into this

**Response:** This has been revised and reported under ‘stillbirth’. Essentially, the IDIs explained the consequences of taking local herbs which was a common practice in some of the study sites. Thus, making it a relevant observation, as a factor associated with the poor outcome of stillbirth, despite the ‘free’ policy.

9. **Comment:** The qualitative findings only talk about the high numbers of stillbirth which have been explained to be caused by poor use of partograph for pregnancy monitoring and shortage of medicines. I highly doubt this is comprehensive and can solely explain the quantitative findings. More exploration of the qualitative data for more reasons to explain the high numbers.

**Response:** Indeed, poor use of partograph as found in the current study is one of the context factor which may be explaining the stillbirth outcomes in the study site. Other context factors that emerged included; late reporting/arrival, poor, shortages of folic acid/multivitamin, and lack of interest in stillbirth ‘matter’. The section has accordingly been revised.

10. **Comment:** “Service providers are of the view that the increase in utilization puts pressure”. Ensure to write the results section in past tense since activities and comments happened sometime back.

**Response:** The results section has been read and revised.

11. **Comment:** On the other hand, pregnant women midwives’ snobbish attitude to them during labour”. Revise sentence as appropriate.

**Response:** sentence has been revised.

12. **Comment:** Just so to confirm are these FGD quotes from mothers that experienced a stillbirth? If so it should be reported as such and if not you need to clarify in the methodology section.

**Response:** Not only mothers who experienced stillbirth, but mothers previous experience while giving birth at the facility level and use of using antenatal care.

13. **Comment:** Due to the facility level shortages, pregnant women regularly asked to purchased medicines outside of the health facility”. Revise statement as appropriate, it is not clear in its current form.

**Response:** Statement revised accordingly.

14. **Comment:** “This was not only a problem to the pregnant women, but also frustrates the quality of processes of the health care professionals” use past tense as appropriate.

**Response:** Manuscript read and edited accordingly.

**Discussion**

1. **Comments:** Revise the discussion section accordingly after effecting changes in the results section

**Response:** Discussion section revised.

2. **Comment:** “improvement in perinatal mortality” is it improvement in perinatal mortality or reduction in perinatal mortality?

**Response:** reduction in perinatal mortality. This has been revised accordingly.

**Conclusion**

1. **Comment:** Revise the discussion section accordingly after effecting changes in the results section

**Response:** Relevant revisions effected, including the conclusion section.

John Azaare (PhD Candidate, Thesis defended successfully)

University for Development Studies

**Lead/Corresponding Author**
