# Supplementary material for: Evaluating the impact of maternal health care policy on stillbirth and perinatal mortality in Ghana; a mixed method approach using two rounds of Ghana demographic and health survey data sets and qualitative design technique
Source: PLoS One. 2022 Sep 29;17(9):e0274573. doi: 10.1371/journal.pone.0274573 (PMC9521900; doi:10.1371/journal.pone.0274573)
Supplement: Attachment — Submitted filename: Reviewer comments_09072022.docx [file pone.0274573.s006.docx]

**Evaluating the impact of maternal health care policy on stillbirth and perinatal mortality in Ghana: a mix method approach using two rounds of Ghana demographic and health survey data sets and qualitative design technique**

Thank you for considering me to review this manuscript. Below are my comments for the authors and editor to consider

**General comments;**

1. The definition of stillbirth should come upfront within the first paragraph of the introductory section. Referring to it as a neglected tragedy is not a definition parse but rather what it is
2. Paragraph two and three are a repetition with one showing absolute numbers and the other showing percentages of global stillbirths. Consider deleting one.
3. The definition of stillbirth is inadequate and its contextual. Rather use the WHO definition which restricts stillbirth to loss after 28 weeks for global comparisons. It is clear that in developed countries the definition can go as low as 20 weeks due to viability but that is not the standard definition.
4. It may not necessarily be history of abortion “ Studies have shown that maternal age, rural/urban area of residence, twin pregnancy, ***history of abortion***” but rather negative pregnancy history. It may be pre-term delivery, miscourage, abortion or even stillbirth. You may consider revising the sentence to bring out this fact.
5. Under contextual definitions, you definition of stillbirth is not appropriate. The standard definition is well known and if any adjustments to the definitions were done in this study, kindly indicate so. Key to the definition is the cut off of 28 weeks and being born with no sign of life. You may need to revise or operationalise what you call “dying within day zero after birth” for which I would highly discourage you from using.
6. **Study design:** Are you sure the mixed methods design was in “ two prongs”?. To me it appears it was sequential mixed methods because you started off with the quantitative secondary data and later the qualitative. If that is the case then revise as appropriate.
7. Statement not clear “who were not necessarily stillborn babies”. Did you want to say the the mothers who participated in the FGDs were not necessarily “mothers” to stillborn babies?. If that is the case then revise as appropriate
8. **Qualitative study setting:** other than giving a geographical description, I would rather you write more about the health systems characteristics especially as they relate to maternal health service access and stillbirth burden or characteristics like to lead mothers to having a stillbirth. Still more about the policy under review would work more compared to giving geographical descriptions. The map can be retained though for visual clarity about the study location.
9. **Inclusion and exclusion criteria:** in the inclusion you say you included women 15-49 years and in exclusion you indicate women below 16 years. Where does that put the ones aged 15 years you indicated under inclusion criteria? Were they included or excluded?
10. Besides exclusion is not the direct opposite of inclusion criteria but that those respondents who met the inclusion criteria in the first place and for some reason were excluded from the sample later such as those that met the inclusion requirements but had missing data for other variables. Revise as appropriate.
11. **Data analysis-qualitative:** how was the code book developed?, who coded the data?, how was quality control ensured?, how did you choose the quote to represent others in the results section?
12. Ethics statements are repeated under “***tools and pre-testing”*** and ***“ethical consideration”.*** Consider removing one to avoid repeating yourself
13. **Results:**  source of data presented in table 1, how is that data from the field for you to label it as “field data”. It is clearly from the DHIMS and should be labelled as such.
14. Quantitative findings. Is data from table 1 not part of the quantitative findings? If so then the sub heading of quantitative findings should be shifted up to include results from table one.
15. Rates of stillbirth increasing between the two timepoints this is a unique finding that needs to be explored more. Why the risk appeared to be more in the free maternal health care policy group also needs to be explored.
16. Although there was an overall decline between the two timepoints, the risk of perinatal mortality was also high in the free maternal healthcare policy compared to the control group. This phenomenon needs to be explored and discussed in relation to the national context. It calls for interrogation of the quality of secondary data used. Otherwise the conclusion would be that the free maternal healthcare policy instead led to and increase in both stillbirth and perinatal mortality.
17. Centrally to what is known, the education level in this study seem to disfavour the outcome variables (that the higher one’s education level the more likely they are to have negative outcomes). This needs to be interrogated too in the context of national maternal and perinatal mortality service delivery.
18. Table 7: group discussion the unit is the focus group. Kindly indicate it and in bracket show the total number of participants in those FGDs which is 44. Revise as appropriate
19. “Antepartum (macerated) stillbirths” these happen before the onset of labour and any stillbirth happening after the onset of labour whether outside the health facility is fresh stillbirth. Revise the sentence as appropriate to reflect that death before arriving at the facility is not the cut off to determine macerated stillbirth.
20. “The reasons for the delay in arriving in the health facility”. What I infer from this and subsequent statements is that the pathway to seeking delivery services has a number of bottlenecks which include actors (Traditional birth attendants) and negative cultural practices (use of local herbs) this needs to come out clearly other than amplifying the “kaligutiem”
21. The effects of data capture may need to be brought into the picture here. The increased number/rates of stillbirth may have been due to improvements in data capture that almost all cases are captured. Needs to be discussed if you think it may have an effect on the observed trends between the two timepoints.
22. “Both stillbirth and perinatal deaths were prevalent among” did you try to explore the role of data capture this might play on the observed outcomes? It may be that proper data capture in the free policy group will show many cases which may be the opposite in the other group.
23. “The findings imply that stillbirth failed to show a decline in 2014”. You may need to highlight the limitations of this study wherein the paper objectives were never the main aim of the policy so there is a likelihood that it may have affected the observed trends.
