# Supplementary material for: Evaluating the impact of maternal health care policy on stillbirth and perinatal mortality in Ghana; a mixed method approach using two rounds of Ghana demographic and health survey data sets and qualitative design technique
Source: PLoS One. 2022 Sep 29;17(9):e0274573. doi: 10.1371/journal.pone.0274573 (PMC9521900; doi:10.1371/journal.pone.0274573)
Supplement: Attachment — Submitted filename: Response to Reviewers.docx [file pone.0274573.s007.docx]

THE ACADEMIC EDITOR

PLOS ONE JOURNAL

Dear Editor,

**RE: Evaluating the impact of maternal health care policy on stillbirth and perinatal mortality in Ghana: a mix method approach using two rounds of Ghana demographic and health survey data sets and qualitative design technique.**

Thank you for reviewing this manuscript. Below are the responses to the issues raised.

**General comments.**

1. The definition of stillbirth should come upfront within the first paragraph of the introductory section. Referring to it as a neglected tragedy is not a definition parse but rather what it is.

**Response:** This has been addressed in Page 1, paragraph 1.

2. Paragraphs two and three are a repetition with one showing absolute numbers and the other showing percentages of global stillbirths. Consider deleting one.

**Response:** Paragraph three has been deleted, while paragraph two revised.

3. The definition of stillbirth is inadequate and its contextual. Rather use the WHO definition which restricts stillbirth to loss after 28 weeks for global comparisons. It is clear that in developed countries the definition can go as low as 20 weeks due to viability but that is not the standard definition.

**Response:** This has been revised in page 4, (under ‘Context definition section).

4. It may not necessarily be history of abortion “Studies have shown that maternal age, rural/urban area of residence, twin pregnancy, ***history of abortion***” but rather negative pregnancy history. It may be pre-term delivery, miscourage, abortion or even stillbirth. You may consider revising the sentence to bring out this fact.

**Response:** “history of abortion” revised appropriately in paragraph four (under ‘conceptual framework’ section).

5. Under contextual definitions, you definition of stillbirth is not appropriate. The standard definition is well known and if any adjustments to the definitions were done in this study, kindly indicate so. Key to the definition is the cut off of 28 weeks and being born with no sign of life. You may need to revise or operationalise what you call “dying within day zero after birth” for which I would highly discourage you from using.

**Response:** This has been revised in page 6 (under context definition).

6. **Study design:** Are you sure the mixed methods design was in “two prongs”? To me it appears it was sequential mixed methods because you started off with the quantitative secondary data and later the qualitative. If that is the case, then revise as appropriate.

**Response:** This has been revised in paragraph one, page 8 (under the ‘design section’).

7. Statement not clear “who were not necessarily stillborn babies”. Did you want to say the mothers who participated in the FGDs were not necessarily “mothers” to stillborn babies? If that is the case, then revise as appropriate.

**Response:** This has been revised in paragraph two, page 8 (under the ‘design section’).

8. **Qualitative study setting:** other than giving a geographical description, I would rather you write more about the health systems characteristics especially as they relate to maternal health service access and stillbirth burden, or characteristics like to lead mothers to having a stillbirth. Still more about the policy under review would work more compared to giving geographical descriptions. The map can be retained though for visual clarity about the study location.

**Response:** The two regions are similar in structure and organization relative to health system characteristics, albeit with different outcomes of stillbirth and facility utilization. This has been reported in the manuscript (Table 1) and captured as Figure 3 (page 15).

9. **Inclusion and exclusion criteria:** in the inclusion you say you included women 15-49 years and in exclusion you indicate women below 16 years. Where does that put the ones aged 15 years you indicated under inclusion criteria? Were they included or excluded?

**Response:** Age “15-49’ as captured in the inclusion criteria is in reference to the age range of the secondary data sets as contained in the DHS survey data sets. However, age 16 years and below were excluded from the in-depth interview in line with the legal age limit for females to have consensual sexual decision, thus, pregnancy. To avoid any confusion for readers, the age range of ‘15-49’ in respect of the Ghana DHS survey data set has been removed under the inclusion criteria section.

10. Besides exclusion is not the direct opposite of inclusion criteria but that those respondents who met the inclusion criteria in the first place and for some reason were excluded from the sample later such as those that met the inclusion requirements but had missing data for other variables. Revise as appropriate.

**Response:** This has been revised under the section ‘Inclusion criteria’.

11. **Data analysis-qualitative:** how was the code book developed? who coded the data? how was quality control ensured? how did you choose the quote to represent others in the results section?

**Response:** The first author coded the data and was supervised by the second, and third author. Codes were deductive (prior to conducting in-depth interviews) based on the study objectives of stillbirth and context factors of service utilization. The second and third author checked for quality of codes, clarity and uniqueness, as supervisors of the first author. Afterwards, common themes were constructed inductively through multiple reading of the transcripts. This has been explained in paragraph on of the ‘qualitative analysis’ section.

12. Ethics statements are repeated under “***tools and pre-testing”*** and ***“ethical consideration”.*** Consider removing one to avoid repeating yourself.

**Response:** The ‘ethical statement’ under the “tools and pre-testing” has been removed.

13. **Results:**  source of data presented in table 1, how is that data from the field for you to label it as “field data”. It is clearly from the DHIMS and should be labelled as such.

**Response:** This has been corrected in Table (page 15).

14. Quantitative findings. Is data from table 1 not part of the quantitative findings? If so then the sub heading of quantitative findings should be shifted up to include results from table one.

**Response:** This comment has been addressed under the ‘Results’ section in page 14.

15. Rates of stillbirth increasing between the two timepoints this is a unique finding that needs to be explored more. Why the risk appeared to be more in the free maternal health care policy group also needs to be explored.

**Response:** We agree with the reviewer. The qualitative aspect of the study was aimed to address this concern, thus, the results as presented in the qualitative section. Of course, further exploration will be useful perhaps in future studies.

16. Although there was an overall decline between the two timepoints, the risk of perinatal mortality was also high in the free maternal healthcare policy compared to the control group. This phenomenon needs to be explored and discussed in relation to the national context. It calls for interrogation of the quality of secondary data used. Otherwise the conclusion would be that the free maternal healthcare policy instead led to an increase in both stillbirth and perinatal mortality.

**Response:** The secondary data used were two-rounds of Ghana DHS data sets (baseline and end line). DHS is a standardly collected data sets using complex design by the DHS Programme, funded by the USAID (independent of this study). The DHS data sets are nationally representative and regularly used in evaluating national programmes or project. Our analysis took clustering and stratification into consideration and applied sample weighting and also adjusted for confounding to minimise internal bias. The study findings of rising stillbirths could perhaps be attributed to increased utilization which may have affected quality of care, and this was corroborated by the qualitative study participants during the IDIs and FGDs.

17. Centrally to what is known, the education level in this study seem to disfavour the outcome variables (that the higher one’s education level the more likely they are to have negative outcomes). This needs to be interrogated too in the context of national maternal and perinatal mortality service delivery.

**Response:** We agree with the reviewers that education appears a disincentive as per the current findings. However, education as reported in this study is a confounding variable. Perhaps, analysing education as the main outcome variable could yield different results, but that is outside the scope of the current study.

18. Table 7: group discussion the unit is the focus group. Kindly indicate it and in bracket show the total number of participants in those FGDs which is 44. Revise as appropriate.

**Response:** This has been revised appropriately in Table 7.

19. “Antepartum (macerated) stillbirths” these happen before the onset of labour and any stillbirth happening after the onset of labour whether outside the health facility is fresh stillbirth. Revise the sentence as appropriate to reflect that death before arriving at the facility is not the cut off to determine macerated stillbirth.

**Response:** This has been revised accordingly in paragraph two, page 27.

20. “The reasons for the delay in arriving in the health facility”. What I infer from this and subsequent statements is that the pathway to seeking delivery services has a number of bottlenecks which include actors (Traditional birth attendants) and negative cultural practices (use of local herbs) this needs to come out clearly other than amplifying the “kaligutiem”

**Response:** Yes, cultural practices such as herbal intake affects the pathway to seeking care during labor. However, our use of the term *‘kalgutiem’* has been chosen to reflect the local context and observations by the service providers. However, this has been revised in pages 29 and 30.

21. The effects of data capture may need to be brought into the picture here. The increased number/rates of stillbirth may have been due to improvements in data capture that almost all cases are captured. Needs to be discussed if you think it may have an effect on the observed trends between the two timepoints.

**Response:** The effect of data capture was brought out by the service providers during the IDIs as reasons for the high figures. The current study did not independently analyse data capture impact to ascertain its influence on the totality of the stillbirth rate. Our analysis of the outcomes of stillbirth based on the secondary data obtained from the DHS programme.

22. “Both stillbirth and perinatal deaths were prevalent among” did you try to explore the role of data capture this might play on the observed outcomes? It may be that proper data capture in the free policy group will show many cases which may be the opposite in the other group.

**Response:** Although this study did not independently analyse the role of data capture, the service providers did mention ‘data capture’ as influencing the high figures during the IDIs, and this has been reported as part of the qualitative findings in page 27, paragraph two and also reflected in the discussion, paragraph nine (page 36).

23. “The findings imply that stillbirth failed to show a decline in 2014”. You may need to highlight the limitations of this study wherein the paper objectives were never the main aim of the policy so there is a likelihood that it may have affected the observed trends.

**Response:** This comment has been addressed in paragraph one under ‘strength and limitation’.

SIGNED

John AZAARE

(Corresponding Author)
